# The impact of commercially available media on cefiderocol susceptibility testing by broth microdilution method

**DOI:** 10.1128/jcm.00471-25

**Published:** 2025-08-20

**Authors:** Boudewijn L.M. DeJonge, Christine Slover, Sean T. Nguyen, Christopher Longshaw, Miki Takemura, Naomi Anan, Hidenori Yamashiro, Yoshinori Yamano

**Affiliations:** 1 Shionogi Inc 71777 Florham Park, New Jersey, USA; 2 Shionogi B.V.London, United Kingdom; 3 Shionogi & Co., LtdOsaka, Japan; Johns Hopkins University, Baltimore, Maryland, USA

**Keywords:** antimicrobial susceptibility testing, broth microdilution, cefiderocol, chelation, Gram-negative species, iron concentration, iron-depleted media

## Abstract

The reference method for determining cefiderocol MIC is broth microdilution (BMD) using iron-depleted cation-adjusted Mueller–Hinton broth (ID-CAMHB). However, laboratories have reported variable MIC findings and difficulties in determining MIC endpoints. To investigate this, the BMD method was re-evaluated by examining the chelation time for iron depletion of the media and by testing reproducibility using four MHB sources (BD-BBL, BD-Difco, Oxoid, and Merck) and 85 Gram-negative isolates (*Escherichia coli*, *Klebsiella pneumoniae*, *Pseudomonas aeruginosa*, and *Acinetobacter baumannii*), of which 63 were previously evaluated *in vivo*. Extending chelation time to 6 h provided optimal iron reduction to ≤0.03 µg/mL for all media. MIC reproducibility was high for each medium source and varied by species (93.3%–99.0% of MIC values within one dilution of modal MIC values); however, different sources of ID-CAMHB produced (reproducible) different modal MIC values for isolates. ID-CAMHB from BD-BBL and BD-Difco showed the best correlation with 96.5% of isolates showing modal MIC values within one dilution between media. MIC values generated with these two media were the most predictive of efficacy *in vivo*. When trailing was observed, it was seen across all media, and refined reading guidelines were applied to enhance reproducibility of determining the MIC endpoint. The 6-h chelation and the refined reading guidelines for trailing are now incorporated into the Clinical and Laboratory Standards Institute M100 document. When using the BMD method to determine cefiderocol susceptibility, the impact of media should be considered, and based on these results, ID-CAMHB prepared with BD-BBL or BD-Difco is recommended.

**IMPORTANCE:**

The reference antibiotic susceptibility testing method for cefiderocol is the broth microdilution method approved by the Clinical and Laboratory Standards Institute in 2016, using iron-depleted cation-adjusted Mueller–Hinton broth. A few manual devices have come to the market replicating this method, but inconsistent results with these devices have been reported. The current study identified the source of Mueller–Hinton broth as a variable in cefiderocol MIC determinations and provides detailed description of the preparation of iron-depleted cation-adjusted Mueller–Hinton broth, and revised reading guidance to improve reproducibility of cefiderocol MIC determinations for *Escherichia coli*, *Klebsiella pneumoniae*, *Pseudomonas aeruginosa*, and *Acinetobacter baumannii*. The recommendations made in this study should enhance the reproducibility of cefiderocol broth microdilution susceptibility testing.

## INTRODUCTION

Cefiderocol is the first-in-class siderophore (catechol) cephalosporin with potent *in vitro* activity against a variety of Gram-negative bacterial species, including carbapenem-resistant isolates of *Acinetobacter baumannii*, *Pseudomonas aeruginosa*, and Enterobacterales (1). The siderophore moiety of cefiderocol mimics natural siderophore molecules released by bacteria to facilitate iron uptake; thus, cefiderocol predominantly enters bacterial cells via active iron transport systems (1–4).

As previously demonstrated for other siderophore antimicrobials (3, 5), *in vitro* susceptibility testing for cefiderocol requires the use of iron-depleted conditions to ensure the expression of iron transporters, and iron-depleted cation-adjusted Mueller–Hinton broth (ID-CAMHB) with iron concentrations of ≤0.03 µg/mL to mimic the *in vivo* conditions of bacteria infecting human tissues and fluids (4, 6). Thus, the reference method for determining cefiderocol MIC is the broth microdilution (BMD) method using ID-CAMHB (6, 7).

Unlike previously developed siderophore antimicrobials, which failed to progress beyond early-stage clinical trials (3), correlation of *in vitro* susceptibility data with *in vivo* effectiveness, without adaptive resistance, was demonstrated for cefiderocol in murine models of Gram-negative infections, including those caused by strains highly resistant to meropenem and cefepime (8, 9).

Concerns have been raised regarding the reproducibility and accuracy of the BMD method for cefiderocol, despite meeting routine quality control (QC) thresholds. There was poor reproducibility of MIC results for *A. baumannii* complex isolates when tested at two different laboratories, with difficulty in interpreting MIC endpoints because of trailing and minor variability in the inoculum preparation resulting in significant differences in cefiderocol MIC values (10). A comment, as well as a testing advisory, was published in the Clinical and Laboratory Standards Institute (CLSI) M100 document, 33rd edition, in 2023, to raise awareness of this issue (10, 11). Shionogi has worked with the CLSI Antimicrobial Susceptibility Testing (AST) subcommittee to resolve these issues and provide further guidance for laboratories (10), which resulted in this manuscript addressing issues using broth microdilution.

Discrepancies in cefiderocol MIC results have been reported when Mueller–Hinton broth (MHB) from different manufacturers is used to prepare ID-CAMHB (4), including the CLSI M23 study, which was used to approve the QC ranges for cefiderocol susceptibility testing in ID-CAMHB (12, 13). The CLSI M23 study already hinted at potential differences in results because of possible disparities in the growth medium (12). The original recommended method for producing the ID-CAMHB medium involved a 2-h chelation time to remove iron cations (4, 6). However, without an accurate measurement of subsequent iron levels, this duration may not have been adequate in some cases to reduce the level to an acceptable target of ≤0.03 µg/mL recommended by the CLSI AST subcommittee and may have contributed to the MIC discrepancies observed across different media (10).

We set out to assess the reproducibility of the BMD method by studying the effect of the chelation time for removal of iron from the medium, and by comparing MIC values obtained with ID-CAMHB prepared from four commercially available sources (i.e., BD-BBL, BD-Difco, Oxoid, Merck) using clinical isolates of *Escherichia coli*, *Klebsiella pneumoniae*, *P. aeruginosa*, and *A. baumannii*.

## MATERIALS AND METHODS

### Bacterial isolates

A total of 85 Gram-negative isolates were obtained from International Health Management Associates (Schaumburg, IL, USA), Eurofins (Luxembourg), American Type Culture Collection (ATCC; Manassas, VA, USA), National Collection of Type Cultures (NCTC; Salisbury, UK), Oita University (Oita, Japan), and by courtesy of Prof. Patrice Nordmann (Fribourg, Switzerland) and Dr. Michael R. Jacobs (Cleveland, OH, USA). The isolates comprised *E. coli* (*n* = 7), *K. pneumoniae* (*n* = 17), *P. aeruginosa* (*n* = 14), and *A. baumannii* (*n* = 47) (Table S1). Among these 85 isolates, 63 (*E. coli n* = 5, *K. pneumoniae n* = 5, *P. aeruginosa n* = 12, *A. baumannii n* = 41) were also tested previously in a murine thigh infection model using humanized cefiderocol exposures (8, 14, 15). CLSI-approved QC strains included *E. coli* ATCC 25922 and *P. aeruginosa* ATCC 27853 (16). Bacterial isolates were identified by matrix-assisted laser desorption/ionization time-of-flight mass spectrometry for the isolates used in the studies of references 8, 14, 15. Information on bacterial identification is not publicly available for the additional isolates.

### Preparation of iron-depleted cation-adjusted Mueller–Hinton broth

ID-CAMHB was prepared as described according to CLSI M100 (16; Video S1). MHB from four manufacturers was tested: BD-BBL Mueller–Hinton II Broth Cation Adjusted (Becton-Dickinson, Sparks, MD, USA; catalog #212322; lot #0252334); BD-Difco Mueller–Hinton Broth (Becton-Dickinson; catalog #275730; lot #1123327); Oxoid Mueller–Hinton Broth (Dehydrated) (ThermoFisher Scientific, Lenexa, KS, USA; catalog #CM0405; lot #3000775); and Merck Mueller–Hinton Broth (Merck KGaA, Darmstadt, Germany; catalog #70192; lot #BCCF7733).

Each MHB was prepared following the respective manufacturer’s instructions. As described previously (6, 17, 18), the iron-depleted medium was prepared by adding 100 g Chelex 100 resin (analytical grade, 100–200 mesh, sodium form; Bio-Rad Laboratories, Hercules, CA, USA) to 1 L of autoclaved MHB. The suspension was stirred with a Teflon-coated magnetic bar for 2 h or 6 h at room temperature (20°C–25°C). The medium was then filtered using a 0.2-µm bottle-top vacuum filter device (Corning Inc., Corning, NY, USA) to remove the resin, and the pH was adjusted to 7.2–7.4 using 6 M hydrochloric acid or 2.5 M sodium hydroxide. Calcium chloride (aimed final concentration of 20–25 µg/mL Ca^2+^), magnesium chloride (aimed final concentration of 10–12.5 µg/mL Mg^2+^), and zinc sulfate (aimed final concentration of 0.5–1.0 µg/mL Zn^2+^) were added to compensate for their removal during the chelation procedure. Again, if needed, the pH was adjusted to 7.2–7.4 using 6 M hydrochloric acid or 2.5 M sodium hydroxide and the broth was filtered using a 0.2-µm bottle-top filter device, as described above. The total iron concentration in each medium was confirmed to be ≤0.03 µg/mL. All stock solutions were prepared with deionized or distilled water. The ID-CAMHB was stored at 4°C–8°C until use for up to 2 months (16).

### Measurement of cation concentrations

The final total iron concentration of ≤0.03 µg/mL was confirmed using a commercial colorimetric test kit, following the manufacturer’s instructions (Visocolor HE Iron, for low concentrations; Macherey-Nagel, Düren, Germany). In addition, the concentration of iron and other cations was measured for each medium using inductively coupled plasma mass spectrometry (ICP-MS) and inductively coupled plasma atomic emission spectrometry (ICP-AES) (Supplementary Methods). The ICP-MS determinations for iron and other cations were performed twice, and average values were calculated.

### Susceptibility testing

Cefiderocol (cefiderocol sulfate tosylate; Shionogi & Co., Ltd, Osaka, Japan) was dissolved and diluted in sterile saline (prepared with deionized water) to create a stock solution of 12.8 mg/mL of free cefiderocol, which was frozen and stored until use. On the day of testing, an aliquot of the frozen cefiderocol stock solution was thawed and diluted 10× with distilled water. From this diluted cefiderocol solution, a twofold dilution series was prepared with distilled water for susceptibility testing, using cluster tubes (Corning Inc.).

Then, 96-well plates were prepared by adding 90 µL of ID-CAMHB, 5 µL of cefiderocol stock solution, and 5 µL of bacterial inoculum according to CLSI guidelines (7, 16). Growth control wells contained ID-CAMHB and bacteria. Sterile control wells contained only ID-CAMHB. The final volume of each well was 100 µL. Susceptibility testing was performed on three different days using three separate inocula (Table S1). On each test day and for each bacterial species, 10 replicates of each isolate (7 *E. coli*, 17 *K*. *pneumoniae*, 14 *P*. *aeruginosa*, and 15 *A*. *baumannii*) were tested in each medium using the same inoculum. Thirty-two additional isolates of *A. baumannii* were tested to expand the data set with more strains of this species that show more complicated MIC read-outs, but these were only tested in triplicate on three different days, given the high reproducibility that was obtained with the initial 53 isolates. For each medium, 210 MIC values were collected for seven *E. coli* isolates, 510 MIC values for 17 *K*. *pneumoniae* isolates, 420 MIC values for 14 *P*. *aeruginosa* isolates, and 738 MIC values for 47 *A*. *baumannii* isolates. QC strains were tested in parallel in each MIC determination.

For inoculation, each bacterial suspension was standardized in saline to a turbidity equivalent to 0.5 McFarland Standard using a spectrophotometer (Ultrospec 6300 Pro, GE Healthcare, Amersham, UK) to reach the optical density of 0.1 at 625 nm (assumed bacterial suspension equivalent to 1 × 10^8^ CFU/mL). The suspension was diluted to an optical density of 0.01 at 625 nm (assumed bacterial suspension equivalent to 1 × 10^7^ CFU/mL). Then, 5 µL of bacterial suspension (final inoculum 5 × 10^5^ CFU/mL) was transferred to each well of the microtiter plates (except the sterile control wells) within 15 min of inoculum preparation.

Isolates were incubated at 35°C for 16–20 h for Enterobacterales and *P. aeruginosa* and for 20–24 h for *A. baumannii* in ambient air before MIC endpoints were read. Susceptibility testing was performed by three experimenters on each day. Test plates were evaluated visually by the naked eye, and MIC values were documented. Photographs of the plates were taken, and these images were used to determine MIC values using the refined CLSI criteria described below (16). In a few cases, re-evaluation led to small discrepancies in the MIC values obtained by the naked eye; however, these were resolved among the experimenters.

Reading of cefiderocol MICs was dependent on the presence of strong growth in the ID-CAMHB growth control wells (i.e., a button size of approximately ≥2 mm or heavy turbidity). The MIC of cefiderocol was determined according to the updated method from the CLSI AST subcommittee (i.e., at the lowest concentration [the first clear well] where no trailing [button ≤1 mm] or light haziness was observed) (16). If reduced growth in cefiderocol-containing wells was observed, the MIC was read as the lowest concentration of cefiderocol in which the reduction of growth compared with the growth control corresponded to a button size of approximately ≤1 mm, or a light haze or faint turbidity with a significant (e.g., ≥80%) reduction compared with the growth control (Video S2) (16).

In cases where a skipped well was observed, the MIC was confirmed at the wells of other replicates without skipped wells.

Susceptibility results were interpreted according to CLSI 2025 M100 breakpoints: susceptible as MIC ≤4 µg/mL; intermediate as 8 µg/mL; resistant as ≥16 µg/mL for Enterobacterales, *P. aeruginosa*, and *A. baumannii* (16).

### Interpretation of test results

Reproducibility of MIC determinations for each specific brand of MHB was assessed for each isolate (number of MIC values within ±1 doubling-dilution from the modal MIC of an isolate), and then summarized for all isolates as the percentage of MIC determinations that fell within ±1 doubling-dilution from the modal MIC values of the isolates. Agreement in MIC values across brands of medium was assessed by comparing the modal MIC values of each isolate obtained with each brand of medium and expressed as the percentage of isolates with modal MIC values within ±1 doubling-dilution across brands.

### Correlation of *in vitro* MIC and *in vivo* efficacy

Modal MIC values obtained in the current study of the isolates, which were evaluated previously *in vivo* in a murine thigh infection model using a humanized dose of cefiderocol (Table S1) (8, 14, 15), were plotted against change in bacterial colony count (i.e., CFU) to determine the correlation between MIC values obtained for each broth source and *in vivo* efficacy. Changes in CFU were taken from the previous murine thigh infection model studies (8, 14, 15), and the original modal MIC values determined with BD-BBL ID-CAMHB in those experiments were also included in the analysis (8).

## RESULTS

### Effect of chelation time on cation content of media

The four different broths were treated for 2 or 6 h with Chelex. The 6-h chelation time resulted in a further reduction of iron content in all media, measured by the Visocolor test kit and ICP-MS (Table 1). While a 2-h chelation time was sufficient to lower the iron concentration to <0.03 µg/mL for BD-BBL, BD-Difco, and Merck MHB, the Oxoid MHB required a 6-h chelation time to reduce the iron content to <0.03 µg/mL. Chelation also removed other cations to negligible levels or below the detection limit, such as magnesium, calcium, and zinc, from all the media tested, but the subsequent additions of calcium, magnesium, and zinc corrected for their removal and provided equal concentrations of these cations across all four media (Table 2). Nickel and aluminum concentrations were also reduced by chelation, but potassium concentration was not (Table S2). The effect on other cations, such as chromium, manganese, cobalt, and copper, was difficult to assess as these are only present in trace amounts and close to the limit of detection of the ICP-AES method.

**TABLE 1:** Iron cation content before and after adjustment in different brands of Mueller–Hinton broth after 0, 2, and 6 h of chelation, as measured using the Visocolor test kit and inductively coupled plasma mass spectrometry (ICP-MS)^*a*^^,^^*b*^^,*e*^

Iron concentration (µg/mL) at baseline and after chelation								
			VisoColor			ICP-MS		
Broth manufacturer/details*^c^*			0 h	2 h*^d^*	6 h*^d^*	0 h	2 h*^d^*	6 h*^d^*
BD-BBL	MHIIB	Before CA	NA	0.01-0.02	≤0.01	NA	**0.038**	0.017
Lot no.	0252334	After CA	**0.15-0.20**	0.02	≤0.01	**0.20**	**0.041**	0.017
BD-Difco	MHB	Before CA	**0.15-0.20**	0.02-0.03	≤0.01	**0.18**	**0.047**	0.025
Lot no.	1123327	After CA	NA	0.02-0.03	≤0.01	NA	**0.047**	0.022
Oxoid	MHB	Before CA	**>0.20**	**0.04-0.05**	0.01-0.02	**0.42**	**0.078**	**0.033**
Lot no.	3000775	After CA	NA	**0.04-0.05**	0.01-0.02	NA	**0.079**	**0.031**
Merck	MHB	Before CA	**0.10**	0.01-0.02	≤0.01	**0.16**	**0.049**	0.027
Lot no.	BCCF7733	After CA	NA	0.01-0.02	≤0.01	NA	**0.049**	0.025

^*a*^ Iron concentrations of >0.03 µg/mL are indicated in bold.

^*b*^ CA, cation adjustment; MHB, Mueller–Hinton broth; MHIIB, Mueller–Hinton II broth; NA, not applicable.

^*c*^ Each cation-adjusted Mueller–Hinton broth (CAMHB) or iron-depleted CAMHB (ID-CAMHB) was prepared from MHB or MHIIB from four different manufacturers: BD-BBL, BD-Difco, Oxoid, Merck.

^*d*^ ID-CAMHB was treated with Chelex for 2 or 6 h, followed by cation (magnesium, calcium, and zinc) adjustment.

^*e*^ ICP-MS values for iron and other cations are the average of two measurements.

**TABLE 2:** Magnesium, calcium, and zinc cation concentrations before and after adjustment in different brands of Mueller–Hinton broth after 0, 2, and 6 h of chelation, as measured using inductively coupled plasma mass spectrometry (ICP-MS)^*a*^^,^^*d*^

			Cation concentration (µg/mL) at baseline and after chelation								
			Mg^2+^			Ca^2+^			Zn^2+^		
Broth manufacturer/details*^b^*			0 h	2 h*^c^*	6 h*^c^*	0 h	2 h*^c^*	6 h*^c^*	0 h	2 h*^c^*	6 h*^c^*
BD-BBL	MHIIB	Before CA	NA	0.04	0.03	NA	0.15	0.15	NA	<0.02	<0.02
Lot no.	0252334	After CA	10	12	11	22	26	24	0.98	0.58	0.57
BD-Difco	MHB	Before CA	4.1	0.01	0.01	3.0	0.08	0.08	0.29	<0.02	<0.02
Lot no.	1123327	After CA	NA	11	11	NA	24	24	NA	0.57	0.56
Oxoid	MHB	Before CA	5.6	0.02	0.02	6.5	0.09	0.09	0.27	<0.02	<0.02
Lot no.	3000775	After CA	NA	11	11	NA	23	24	NA	0.56	0.56
Merck	MHB	Before CA	2.1	0.01	0.01	7.6	0.07	0.07	0.70	<0.02	<0.02
Lot no.	BCCF7733	After CA	NA	11	11	NA	23	23	NA	0.58	0.58

^*a*^ CA, cation adjustment; MHB, Mueller–Hinton broth; MHIIB, Mueller–Hinton II broth; NA, not applicable.

^*b*^ Each cation-adjusted Mueller–Hinton broth (CAMHB) or iron-depleted CAMHB (ID-CAMHB) was prepared from MHB or MHIIB from four different manufacturers: BD-BBL, BD-Difco, Oxoid, Merck.

^*c*^ ID-CAMHB was treated with Chelex for 2 or 6 h, followed by cation (magnesium, calcium, and zinc) adjustment.

^*d*^ ICP-MS values for iron and other cations are the average of two measurements.

### Media effect on MIC values

The MIC values for the *E. coli*, *K. pneumoniae*, *P. aeruginosa*, and *A. baumannii* isolates were determined with ID-CAMHB prepared from four MHB sources using the 6-h chelation time (Table S1).

MICs were highly reproducible within single plates and across 3 days of testing for each respective ID-CAMHB (Table 3; Table S1). Reproducible MIC values were obtained for each bacterial species for each ID-CAMHB, with ≥93.3% of the MIC values being within one doubling-dilution from the modal MIC of that brand of medium. However, there was a variation in MIC values observed between ID-CAMHB sourced from different manufacturers (Table 4). MIC values obtained with ID-CAMHB sourced from BD-BBL and BD-Difco showed the best reproducibility, with 96.5% of the modal MIC values of the isolates being within one dilution. Modal MIC values obtained with ID-CAMHB from BD-Difco and Oxoid were within one dilution for 92.9% of the isolates, but for other media comparisons, the percentages were lower (58.8%–80.0%). The MIC discrepancies were observed across MIC values and species (Table S1).

**TABLE 3:** Percentage of MIC agreement within a brand of iron-depleted cation-adjusted Mueller–Hinton broth after 6-h chelation time

	Percentage MIC agreement within broth manufacturer sources											
	*E. coli^a^*			*K. pneumoniae^b^*			*P. aeruginosa^c^*			*A. baumannii^d^*		
	±1 dilution*^e^*	±2 dilution*^f^*	± ≥ 3 dilution*^g^*	±1 dilution*^e^*	±2 dilution*^f^*	± ≥ 3 dilution*^g^*	±1 dilution*^e^*	±2 dilution*^f^*	± ≥ 3 dilution*^g^*	±1 dilution*^e^*	±2 dilution*^f^*	± ≥ 3 dilution*^g^*
BD-BBL	99.0	1.0	0.0	94.5	2.9	2.5	93.3	6.7	0.0	94.4	3.8	1.8
BD-Difco	97.6	2.4	0.0	95.5	2.7	1.8	96.2	3.8	0.0	96.1	3.3	0.7
Oxoid	97.1	2.9	0.0	97.6	2.4	0.0	96.7	1.2	2.1	96.1	2.2	1.8
Merck	98.5	1.0	0.5	95.1	1.6	3.3	95.2	4.5	0.2	96.1	2.2	1.8

^*a*^ 7 isolates and 210 MIC values/medium.

^*b*^ 17 isolates and 510 MIC values/medium.

^*c*^ 14 isolates and 420 MIC values/medium.

^*d*^ 47 isolates and 738 MIC values/medium.

^*e*^ Percentage of MIC values with ±1 dilution of modal MIC of isolates.

^*f*^ Percentage of MIC values with ±2 dilutions of modal MIC of isolates.

^*g*^ Percentage of MIC values with ±≥3 dilutions of modal MIC of isolates.

**TABLE 4:** Percentage of MIC agreement across the four brands of iron-depleted cation-adjusted Mueller–Hinton broth after 6 h chelation time

Percentage agreement of MIC values across broth manufacturer sources*^a^*			
Broth manufacturer comparison	±1 dilution*^b^*	±2 dilution*^c^*	± ≥ 3 dilution*^d^*
BD-BBL vs BD-Difco	96.5	2.4	1.2
BD-BBL vs Oxoid	80.0	11.8	8.2
BD-BBL vs Merck	62.4	11.8	25.9
BD-Difco vs Oxoid	92.9	4.7	2.4
BD-Difco vs Merck	58.8	21.2	20.0
Oxoid vs Merck	60.0	11.8	28.2

^*a*^ 85 isolates and 1,878 MIC values/medium.

^*b*^ Percentage of isolates with modal MIC values with ±1 dilution.

^*c*^ Percentage of isolates with modal MIC values with ±2 dilutions.

^*d*^ Percentage of isolates with modal MIC values with ±≥3 dilutions.

This phenomenon was illustrated With *E. coli* EC461 and *K. pneumoniae* KP532, which showed highly reproducible MIC values on the 3 days of testing for each medium but discrepant MIC values across the media (Fig. S1A and B). *E. coli* EC466 and *P. aeruginosa* PA1562 are examples of isolates that showed some variability on each day of testing but consistent MIC values across the media (Fig. S1C and D). The abovementioned three Enterobacterales isolates all produced distinct MIC endpoints, with obvious suppression of growth, but this was not the case for *P. aeruginosa* PA1562 and PA1568, where some skipped wells were observed (Fig. S1D and E). Skipped wells were not reproducible and were ignored in these experiments because 10 replicates of the strains were tested, and therefore, the growth observed in lanes without skipped wells could be used to guide the MIC value. In addition, trailing was observed for some isolates, as illustrated for *K. pneumoniae* KP549 (Fig. S1F), but this was most apparent for several *A. baumannii* isolates (Fig. S1G and H). Trailing was independent of media and was reproducible across the days as shown for *A. baumannii* AB126 and AB NCTC13301 (Fig. S1G and H). Application of refined reading guidance allowed for more consistent MIC read-out across experimenters. However, despite applying the revised CLSI reading guidance (16), trailing led to discrepant MIC endpoints still being recorded, showing that subjective assessment of the MIC endpoint still remains a possibility. Increased growth (or regrowth) was at times also observed at the end of trailing (Fig. S1F and G). In an effort to standardize the MIC read-out when trailing was observed, the MIC was read at the lowest concentration at which a significant reduction in growth (≥80%) was apparent compared with the growth control, or where the button size was ≤1 mm (Video S2). Finally, an unusual growth reduction, in the shape of a shattered star or a donut, was observed for some isolates (e.g., *A. baumannii* strain AB148) (Fig. S1I), and this phenomenon was reproducible across days and media. The MIC was read at the lowest concentration of cefiderocol at which growth/haze was significantly reduced (≥80% reduction) compared with the growth control.

### Correlation between *in vitro* and *in vivo* results

To understand which MIC value is most predictive of *in vivo* efficacy, modal MIC values obtained with the different media were plotted against the change in bacterial colony counts obtained in the mouse thigh infection model (8). For susceptible isolates, a reduction in bacterial load is expected upon treatment, whereas for resistant isolates, an increase in bacterial load is expected. Isolates with intermediate susceptibility (i.e., MIC value of 8 µg/mL) were excluded from the calculations as their results in the animal model could be ambiguous.

**Fig 1:**
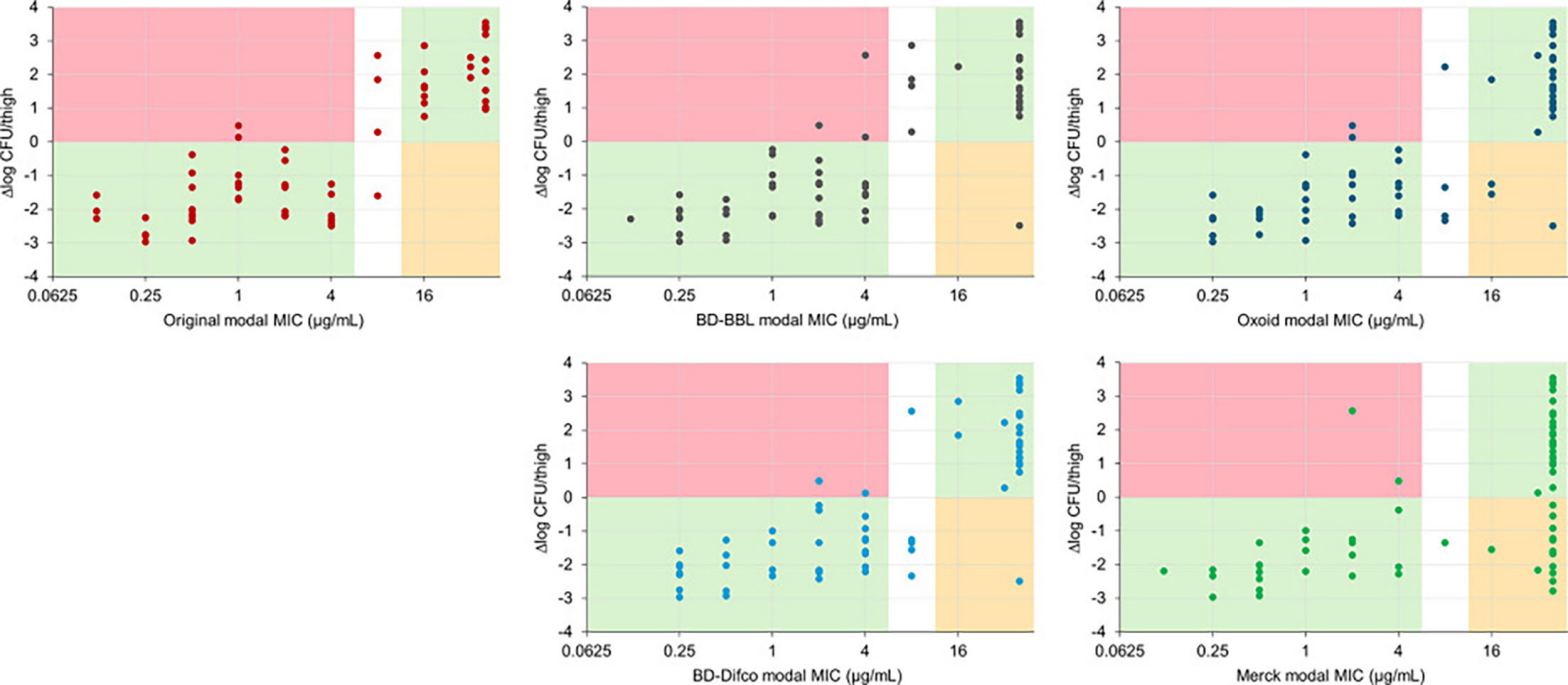
Correlation between *in vitro* MIC (µg/mL) of cefiderocol determined in iron-depleted cation-adjusted Mueller–Hinton broth after 6-h chelation of different sources of broth and response in *in vivo* efficacy (∆log CFU/thigh) for 63 isolates that were tested in a murine thigh infection model. Pink area: includes isolates for which modal MIC value is in the susceptible range but CFU change was positive; orange area: includes isolates for which modal MIC is in the resistant range but CFU change was negative; green area: includes isolates for which modal MIC values correlate with *in vivo* CFU change. White area: includes isolates for which modal MIC is in the intermediate range, whose results in the animal model could be ambiguous. Original MIC and CFU data adapted from references 8, 14, 15.

Modal MIC values determined in ID-CAMHB prepared from BD-BBL and BD-Difco broths were highly predictive of *in vivo* efficacy, with modal MIC values for 93.2% (55/59) and 94.8% (55/58) of the isolates, respectively, correctly reflecting decreases or increases in CFU in the murine thigh infection model (Fig. 1). With ID-CAMHB prepared from BD-BBL broth, three of 39 susceptible isolates (two *A. baumannii* and one *P. aeruginosa*) showed an increase in CFU *in vivo,* and one of 20 resistant isolates (*A. baumannii*) showed a decrease in CFU *in vivo*. For ID-CAMHB prepared with BD-Difco broth, two of 34 susceptible isolates (same *A. baumannii* as for BD-BBL) showed a CFU increase, and one of 24 resistant isolates (same *A. baumannii* as for BD-BBL) showed a CFU decrease (Fig. 1). For the Oxoid broth, three of 29 resistant isolates (one *E. coli*, one *K. pneumoniae*, and the same *A. baumannii* as for BD-BBL and BD-Difco) showed CFU decreases, but for the Merck broth, 13 of 37 resistant isolates showed CFU decreases; overall agreements between *in vitro* and *in vivo* results were 91.5% and 75.8% for Oxoid and Merck broths, respectively.

## DISCUSSION

The current study sought to address discrepancies reported with cefiderocol BMD susceptibility testing (4, 10). The first parameter assessed was the chelation time of the test medium. Initially, a 2-h period of chelation was recommended (6), but the current study showed that this chelation time was not sufficient to deplete iron consistently in different sources of media. Insufficient iron depletion could lead to more elevated MIC values and to less reproducibility when MIC values are determined with the BMD method using ID-CAMHB from different sources. We showed that extending the chelation time to 6 h removed iron from all media sources to levels of ≤0.03 µg/mL (19). The low iron content was confirmed with Visocolor, a semi-quantitative measurement of free iron using a color-based triazine technique, as well as with ICP-MS, which gives an elemental measurement of both free and bound iron. As chelation of the media also removed other cations, the replenishment of essential bivalent cations needed for growth, such as calcium, magnesium, and zinc, was necessary. The optimized conditions for the preparation of ID-CAMHB have been incorporated into the latest CLSI performance standards for cefiderocol susceptibility testing (Video S1; Fig. S2) (16).

The second parameter assessed to explain discrepancies reported with cefiderocol BMD susceptibility testing was the reproducibility of MIC determination with different ID-CAMHB prepared according to the new procedure. Reproducible cefiderocol MIC values were obtained for the isolates with each tested medium (i.e., >93% per species). Across the four media sources, the highest reproducibility was found for *E. coli* (i.e., 97.1%–99.0%), while reproducibility ranged between 94.5% and 97.6% for *K. pneumoniae*, between 93.3% and 96.7% for *P. aeruginosa*, and between 94.4% and 96.1% for *A. baumannii* (Table 3). However, differences were observed in the MIC values obtained with ID-CAMHB prepared from the different brands of MHB. The highest agreement in modal MIC values (±1 dilution) was found between media from BD-BBL and BD-Difco and between media from BD-Difco and Oxoid, but for other media, the agreements were much lower. These differences across the media could not be attributed to the iron content, which was uniformly depleted to a concentration of ≤0.03 µg/mL. A limitation of this study was that only one lot of each media brand was used in this study; thus, reproducibility across media lots of one brand was not studied. However, limited data with 23 isolates on reproducibility of MIC values in ID-CAMHB using three lots of BD-BBL and BD-Difco and two lots of Oxoid media showed good reproducibility for the different lots of media (unpublished data).

Discrepancies were observed across the species. For example, *E. coli* EC461 showed clear and reproducible MIC endpoints, yet the MIC values varied across the media sources between 0.5 and 4 µg/mL. Although trailing makes it difficult to determine MIC endpoints and can therefore contribute to MIC discrepancies, this was a less concerning factor in the current study as all the experiments were performed in one laboratory using refined reading guidance to determine the MIC endpoints (Video S2). Despite standardizing the read-out of MICs for strains showing trailing, subjectivity on how to interpret the size of the button and the magnitude of growth reduction will remain, affecting reproducibility of MIC read-outs, especially across technicians and laboratories, for isolates that show trailing. No clear pattern was found in relation to species, MIC value, molecular characteristics, or beta-lactamase expression that could explain these differences. It is unclear what causes these MIC discrepancies, but it is possible that, beyond iron, other unidentified factors related to the medium regulate the expression of iron uptake systems and hence activity of cefiderocol.

Issues with susceptibility testing of (primarily) *Acinetobacter* spp. have been reported for antibiotics other than cefiderocol. Swenson et al. reported problems with the accurate interpretation of MICs for beta-lactams – but not for other classes of antibiotics – using the BMD method (20). Specifically, subtle bacterial growth (described as granular, a small button, or star-like) was observed beyond an obvious endpoint, similarly to our observations with cefiderocol for the *A. baumannii* AB148 strain. Other reports have subsequently been published on issues regarding MIC interpretations for carbapenems and colistin when testing *Acinetobacter* spp. and *K. pneumoniae* isolates, with methods failing to meet the criteria for acceptable antimicrobial susceptibility test performance (21, 22). There was also major variation in MICs determined for tigecycline specifically against *A. baumannii*, which depended on the method used (i.e., E-test or BMD), and which was not evident for other species tested (23). In addition, a discrepancy in MIC values for minocycline has been reported against carbapenem-resistant strains of *A. baumannii* using regular (cation-unadjusted) MHB sourced from different manufacturers (BD and Oxoid) (24). Other environmental factors that affect the MIC of antibiotics include pretreatment of microtiter plates to influence adherence of polymyxins, such as colistin, to the plate surface (22). Additionally, different concentrations of various cations in the media may impact MIC values obtained by BMD methods; for example, concentrations of Zn^2+^ cation have an impact on imipenem susceptibility of *P. aeruginosa* or *S. maltophilia* (25–27) or on meropenem susceptibility of Enterobacterales that produce metallo-beta-lactamases (28). Of note, Mn^2+^ concentration in the medium may impact tigecycline susceptibility in extended-spectrum beta-lactamase-producing Enterobacterales or *A. baumannii* (29). Additional studies are required to understand which environmental factor(s) in the medium, beyond iron, can affect the MIC of cefiderocol and other antibiotics. Once identified, all media could be standardized accordingly.

Given the discrepancy in MIC values when using ID-CAMHB prepared from different brands of MHB, the reported lack of agreement in cefiderocol MIC values obtained with commercially available test kits should not be surprising if different base broths for ID-CAMHB were used for the commercial and the reference methods. Thus, Kolesnik-Goldmann et al. used Merck MHB and a 2-h chelation time in the preparation of ID-CAMHB (reference BMD method) and compared the performance of this method with that of the UMIC Cefiderocol assay (Bruker Daltonics GmbH & Co. KG, Bremen, Germany) and the ComASP cefiderocol microdilution panel (Liofilchem, Roseto degli Abruzzi, Italy), two BMD assays using pre-prepared ID-CAMHB from the manufacturers. The authors found only 76% essential agreement with the reference BMD method for both UMIC and ComASP (30). Similarly, Jeannot et al. (31) tested cefiderocol susceptibility of *Acinetobacter* spp. by comparing the performance of UMIC and ComASP versus the BMD reference method, in which BD broth was used to prepare ID-CAMHB. Both UMIC and ComASP methods achieved an essential agreement of only around 80% in this study (31). Similarly, when MIC values obtained with the ComASP and UMIC assays were compared with those obtained from frozen panels prepared by ThermoFisher (Sensititre CML1FEUD plate), essential agreements for UMIC and ComASP were 91.7% and 76.7%, respectively, for a challenging collection of highly resistant Enterobacterales (32). If the pre-prepared ID-CAMHB from the UMIC and ComASP assays is not based on Merck, BD, or ThermoFisher broths, these discrepancies are not surprising given the results of our studies. Much higher essential agreement was observed when reference ID-CAMHB was used from the same manufacturer. Thus, the essential agreement between BMD using ID-CAMHB from Bruker, the manufacturer of UMIC, and the UMIC assay was >90% in a study by Dortet et al. using 283 Gram-negative isolates, and 85.5% in a study by Bianco et al. on a collection of 256 isolates (33, 34).

MIC values are important because they can be used to predict efficacy *in vivo* and guide appropriate use of antibiotics with *in vitro* activity. Given the variability of MIC values obtained with the different ID-CAMHBs, we analyzed which media would best predict *in vivo* effectiveness based on those MIC values and hence are more relevant for susceptibility testing. MIC values measured in ID-CAMHB prepared with BD-BBL and BD-Difco broths were the most predictive for *in vivo* outcomes in the mouse thigh infection model. A few discrepancies were observed between the *in vitro* MIC values determined using either BD-BBL or BD-Difco ID-CAMHB and the *in vivo* response, in line with historical data that were used to set breakpoints, while more frequent discrepancies were found between *in vitro* MIC values determined using ID-CAMHB prepared with Merck and Oxoid broths. Therefore, it is recommended that MIC BMD testing for cefiderocol is performed in ID-CAMHB prepared using MHB BD-Difco or MHIIB BD-BBL.

### Conclusions

This study identified the need for a longer (6 h) chelation time during the preparation of ID-CAMHB used in susceptibility testing for cefiderocol, to ensure an optimally low iron concentration, especially when using different media sources. This 6-h chelation step for iron depletion has now been incorporated into the CLSI methodology for preparation of ID-CAMHB. Despite the uniform iron depletion across all media, different MIC values were still obtained across different broths. The reason for this is currently unclear, but together with the insufficient iron depletion that may have occurred with the 2-h chelation, these media discrepancies probably contribute to the disagreements reported in the literature on cefiderocol susceptibility testing. The highest concordance of MIC values between the different commercial manufacturers’ media was found between BD-BBL and BD-Difco, and MIC values obtained with these media were also the most predictive for *in vivo* response. Therefore, it is recommended that cefiderocol susceptibility testing is performed with ID-CAMHB prepared from BD-BBL or BD-Difco MHB.

## Data Availability

All analyzed data are included in the manuscript or supplemental materials.

## SUPPLEMENTAL MATERIAL

Fig. S1 jcm.00471-25-s0001.pdf MIC determinations for *E. coli, K. pneumoniae, P. aeruginosa*, and *A. baumannii* strains using ID-CAMHB sourced from BD-BBL, BD-Difco, Oxoid, and Merck.

Fig. S2 jcm.00471-25-s0002.pdf Summary of the preparation of the iron-depleted cation-adjusted Mueller–Hinton broth.

Supplemental Methods jcm.00471-25-s0003.pdf Protocol of inductively coupled plasma atomic emission spectrometry (ICP-AES) and inductively coupled plasma mass spectrometry (ICP-MS).

Table S1 jcm.00471-25-s0004.xlsx MIC values for all isolates measured in iron-depleted cation-adjusted Mueller–Hinton broth prepared from four brands of media with a 6-h chelation time.

Table S2 jcm.00471-25-s0005.pdf Unadjusted cation concentrations in different brands of cation-adjusted Mueller–Hinton broth measured using ICP-AES after 0 and 24 h of chelation.

Video S1 jcm.00471-25-s0006.mp4 Preparation of iron-depleted cation-adjusted Mueller–Hinton broth.

Video S2 jcm.00471-25-s0007.mp4 Determination of cefiderocol MIC values using the broth microdilution method.

ASM does not own the copyrights to Supplemental Material that may be linked to, or accessed through, an article. The authors have granted ASM a non-exclusive, world-wide license to publish the Supplemental Material files. Please contact the corresponding author directly for reuse.

## References

[1] Sato T, Yamawaki K. 2019. Cefiderocol: discovery, chemistry, and in vivo profiles of a novel siderophore cephalosporin. Clin Infect Dis 69:S538–S543. doi:10.1093/cid/ciz826 31724047 PMC6853759

[2] Ito A, Sato T, Ota M, Takemura M, Nishikawa T, Toba S, Kohira N, Miyagawa S, Ishibashi N, Matsumoto S, Nakamura R, Tsuji M, Yamano Y. 2018. In vitro antibacterial properties of cefiderocol, a novel siderophore cephalosporin, against gram-negative bacteria. Antimicrob Agents Chemother 62:e01454-17. doi:10.1128/AAC.01454-17 PMC5740388 29061741

[3] Page MGP. 2019. The role of iron and siderophores in infection, and the development of siderophore antibiotics. Clin Infect Dis 69:S529–S537. doi:10.1093/cid/ciz825 31724044 PMC6853763

[4] Hackel MA, Tsuji M, Yamano Y, Echols R, Karlowsky JA, Sahm DF. 2019. Reproducibility of broth microdilution MICs for the novel siderophore cephalosporin, cefiderocol, determined using iron-depleted cation-adjusted Mueller-Hinton broth. Diagn Microbiol Infect Dis 94:321–325. doi:10.1016/j.diagmicrobio.2019.03.003 31029489

[5] Luscher A, Moynié L, Auguste PS, Bumann D, Mazza L, Pletzer D, Naismith JH, Köhler T. 2018. TonB-dependent receptor repertoire of Pseudomonas aeruginosa for uptake of siderophore-drug conjugates. Antimicrob Agents Chemother 62:e00097-18. doi:10.1128/AAC.00097-18 29555629 PMC5971595

[6] Hackel MA, Tsuji M, Yamano Y, Echols R, Karlowsky JA, Sahm DF. 2017. In vitro activity of the siderophore cephalosporin, cefiderocol, against a recent collection of clinically relevant gram-negative bacilli from North America and Europe, including carbapenem-nonsusceptible isolates (SIDERO-WT-2014 Study). Antimicrob Agents Chemother 61:e00093-17. doi:10.1128/AAC.00093-17 28630181 PMC5571285

[7] Clinical and Laboratory Standards Institute (CLSI). 2024. Methods for dilution antimicrobial susceptibility tests for bacteria that grow aerobically. In CLSI standard M07, 12th ed. CLSI, Wayne, PA, USA.

[8] Monogue ML, Tsuji M, Yamano Y, Echols R, Nicolau DP. 2017. Efficacy of humanized exposures of cefiderocol (S-649266) against a diverse population of gram-negative bacteria in a murine thigh infection model. Antimicrob Agents Chemother 61:e01022–17. doi:10.1128/AAC.01022-17 28848004 PMC5655050

[9] Ghazi IM, Monogue ML, Tsuji M, Nicolau DP. 2018. Humanized exposures of cefiderocol, a siderophore cephalosporin, display sustained in vivo activity against siderophore-resistant Pseudomonas aeruginosa. Pharmacology 101:278–284. doi:10.1159/000487441 29471305 PMC5972512

[10] Simner PJ, Palavecino EL, Satlin MJ, Mathers AJ, Weinstein MP, Lewis JS II, Humphries R. 2023. Potential of inaccurate cefiderocol susceptibility results: a CLSI AST subcommittee advisory. J Clin Microbiol 61:e0160022. doi:10.1128/jcm.01600-22 36946754 PMC10117095

[11] Clinical and Laboratory Standards Institute (CLSI). 2023. Performance standards for antimicrobial susceptibility testing. In CLSI Supplement M100, 33rd ed. CLSI, Wayne, PA, USA.

[12] Huband MD, Ito A, Tsuji M, Sader HS, Fedler KA, Flamm RK. 2017. Cefiderocol MIC quality control ranges in iron-depleted cation-adjusted Mueller-Hinton broth using a CLSI M23-A4 multi-laboratory study design. Diagn Microbiol Infect Dis 88:198–200. doi:10.1016/j.diagmicrobio.2017.03.011 28410852

[13] Clinical and Laboratory Standards Institute. 2023. Development of *in vitro* susceptibility testing criteria and quality control parameters. In M23-A6, 6th ed. CLSI, Wayne, PA, USA.

[14] Gill CM, Abdelraouf K, Oota M, Nakamura R, Kuroiwa M, Ishioka Y, Takemura M, Yamano Y, Nicolau DP. 2022. Assessment of sustained efficacy and resistance emergence under human-simulated exposure of cefiderocol against Acinetobacter baumannii using in vitro chemostat and in vivo murine infection models. JAC Antimicrob Resist 4:dlac047. doi:10.1093/jacamr/dlac047 35529054 PMC9070809

[15] Gill CM, Santini D, Takemura M, Longshaw C, Yamano Y, Echols R, Nicolau DP. 2023. In vivo efficacy & resistance prevention of cefiderocol in combination with ceftazidime/avibactam, ampicillin/sulbactam or meropenem using human-simulated regimens versus Acinetobacter baumannii. J Antimicrob Chemother 78:983–990. doi:10.1093/jac/dkad032 36775993 PMC10068413

[16] Clinical and Laboratory Standards Institute (CLSI). 2025. Performance standards for antimicrobial susceptibility testing. In Supplement M100, 35th ed. CLSI, Wayne, PA, USA.

[17] Karlowsky JA, Hackel MA, Tsuji M, Yamano Y, Echols R, Sahm DF. 2019. In vitro activity of cefiderocol, a siderophore cephalosporin, against gram-negative bacilli isolated by clinical laboratories in North America and Europe in 2015-2016: SIDERO-WT-2015. Int J Antimicrob Agents 53:456–466. doi:10.1016/j.ijantimicag.2018.11.007 30471402

[18] Kazmierczak KM, Tsuji M, Wise MG, Hackel M, Yamano Y, Echols R, Sahm DF. 2019. In vitro activity of cefiderocol, a siderophore cephalosporin, against a recent collection of clinically relevant carbapenem-non-susceptible Gram-negative bacilli, including serine carbapenemase- and metallo-β-lactamase-producing isolates (SIDERO-WT-2014 Study). Int J Antimicrob Agents 53:177–184. doi:10.1016/j.ijantimicag.2018.10.007 30395986

[19] Ito A, Nishikawa T, Matsumoto S, Yoshizawa H, Sato T, Nakamura R, Tsuji M, Yamano Y. 2016. Siderophore cephalosporin cefiderocol utilizes ferric iron transporter systems for antibacterial activity against Pseudomonas aeruginosa. Antimicrob Agents Chemother 60:7396–7401. doi:10.1128/AAC.01405-16 27736756 PMC5119021

[20] Swenson JM, Killgore GE, Tenover FC. 2004. Antimicrobial susceptibility testing of Acinetobacter spp. by NCCLS broth microdilution and disk diffusion methods. J Clin Microbiol 42:5102–5108. doi:10.1128/JCM.42.11.5102-5108.2004 15528702 PMC525209

[21] Markelz AE, Mende K, Murray CK, Yu X, Zera WC, Hospenthal DR, Beckius ML, Calvano T, Akers KS. 2011. Carbapenem susceptibility testing errors using three automated systems, disk diffusion, Etest, and broth microdilution and carbapenem resistance genes in isolates of Acinetobacter baumannii-calcoaceticus complex. Antimicrob Agents Chemother 55:4707–4711. doi:10.1128/AAC.00112-11 21807971 PMC3187004

[22] Dafopoulou K, Zarkotou O, Dimitroulia E, Hadjichristodoulou C, Gennimata V, Pournaras S, Tsakris A. 2015. Comparative evaluation of colistin susceptibility testing methods among carbapenem-nonsusceptible Klebsiella pneumoniae and Acinetobacter baumannii clinical isolates. Antimicrob Agents Chemother 59:4625–4630. doi:10.1128/AAC.00868-15 26014928 PMC4505270

[23] Marchaim D, Pogue JM, Tzuman O, Hayakawa K, Lephart PR, Salimnia H, Painter T, Zervos MJ, Johnson LE, Perri MB, Hartman P, Thyagarajan RV, Major S, Goodell M, Fakih MG, Washer LL, Newton DW, Malani AN, Wholehan JM, Mody L, Kaye KS. 2014. Major variation in MICs of tigecycline in Gram-negative bacilli as a function of testing method. J Clin Microbiol 52:1617–1621. doi:10.1128/JCM.00001-14 24599978 PMC3993642

[24] Wang P, Bowler SL, Kantz SF, Mettus RT, Guo Y, McElheny CL, Doi Y. 2016. Comparison of minocycline susceptibility testing methods for carbapenem-resistant Acinetobacter baumannii. J Clin Microbiol 54:2937–2941. doi:10.1128/JCM.01810-16 27629901 PMC5121382

[25] Cooper GL, Louie A, Baltch AL, Chu RC, Smith RP, Ritz WJ, Michelsen P. 1993. Influence of zinc on Pseudomonas aeruginosa susceptibilities to imipenem. J Clin Microbiol 31:2366–2370. doi:10.1128/jcm.31.9.2366-2370.1993 8408557 PMC265762

[26] Daly JS, Dodge RA, Glew RH, Soja DT, DeLuca BA, Hebert S. 1997. Effect of zinc concentration in Mueller-Hinton agar on susceptibility of Pseudomonas aeruginosa to imipenem. J Clin Microbiol 35:1027–1029. doi:10.1128/jcm.35.4.1027-1029.1997 9157125 PMC229730

[27] Hawkey PM, Birkenhead D, Kerr KG, Newton KE, Hyde WA. 1993. Effect of divalent cations in bacteriological media on the susceptibility of Xanthomonas maltophilia to imipenem, with special reference to zinc ions. J Antimicrob Chemother 31:47–55. doi:10.1093/jac/31.1.47 8444674

[28] Bilinskaya A, Buckheit DJ, Gnoinski M, Asempa TE, Nicolau DP. 2020. Variability in zinc concentration among Mueller-Hinton broth brands: impact on antimicrobial susceptibility testing of metallo-β-lactamase-producing Enterobacteriaceae J Clin Microbiol 58:e02019-20. doi:10.1128/JCM.02019-20 32999009 PMC7685897

[29] Fernández-Mazarrasa C, Mazarrasa O, Calvo J, del Arco A, Martínez-Martínez L. 2009. High concentrations of manganese in Mueller-Hinton agar increase MICs of tigecycline determined by Etest. J Clin Microbiol 47:827–829. doi:10.1128/JCM.02464-08 19144806 PMC2650928

[30] Kolesnik-Goldmann N, Seth-Smith HMB, Haldimann K, Imkamp F, Roloff T, Zbinden R, Hobbie SN, Egli A, Mancini S. 2023. Comparison of disk diffusion, E-Test, and broth microdilution methods for testing in vitro activity of cefiderocol in Acinetobacter baumannii. Antibiotics (Basel) 12:1212. doi:10.3390/antibiotics12071212 37508308 PMC10376138

[31] Jeannot K, Gaillot S, Triponney P, Portets S, Pourchet V, Fournier D, Potron A. 2023. Performance of the disc diffusion method, MTS gradient tests and two commercially available microdilution tests for the determination of cefiderocol susceptibility in Acinetobacter spp. Microorganisms 11:1971. doi:10.3390/microorganisms11081971 37630529 PMC10458114

[32] Emeraud C, Gonzalez C, Dortet L. 2023. Comparison of ComASP and UMIC methods with the reference method for cefiderocol susceptibility testing on carbapenem-resistant Enterobacterales. J Antimicrob Chemother 78:1800–1801. doi:10.1093/jac/dkad134 37141286

[33] Dortet L, Niccolai C, Pfennigwerth N, Frisch S, Gonzalez C, Antonelli A, Giani T, Hoenings R, Gatermann S, Rossolini GM, Naas T. 2023. Performance evaluation of the UMIC Cefiderocol to determine MIC in Gram-negative bacteria. J Antimicrob Chemother 78:1672–1676. doi:10.1093/jac/dkad149 37209112 PMC10320108

[34] Bianco G, Boattini M, Comini S, Gaibani P, Cavallo R, Costa C. 2024. Performance evaluation of Bruker UMIC microdilution panel and disc diffusion to determine cefiderocol susceptibility in Enterobacterales, Acinetobacter baumannii, Pseudomonas aeruginosa, Stenotrophomonas maltophilia, Achromobacter xylosoxidans and Burkolderia species. Eur J Clin Microbiol Infect Dis 43:559–566. doi:10.1007/s10096-024-04745-7 38240988

