# Supplementary material for: The impact of commercially available media on cefiderocol susceptibility testing by broth microdilution method
Source: J Clin Microbiol. 2025 Aug 20;63(9):e00471-25. doi: 10.1128/jcm.00471-25 (PMC12421808; doi:10.1128/jcm.00471-25)

Supplemental FIG S1(A)–S1(I) MIC determinations for *E. coli*, *K. pneumoniae*, *P. aeruginosa*, and *A. baumannii* strains using iron-depleted cation-adjusted Mueller–Hinton broth (ID-CAMHB) sourced from BD-BBL, BD-Difco, Oxoid, and Merck. ID-CAMHB was prepared with a 6-h chelation time.

Footnotes:

(A) EC461: This strain illustrates MIC reproducibility across wells and days for each medium but shows different MIC values between sources of broth. MIC read-out is clear, with no trailing or ambiguity.
(B) KP532: This strain illustrates variability within one dilution for some media, but the same MIC values across days for each medium. MIC values are different for different media. MIC read-out is clear, with no trailing or ambiguity.
(C) EC466: This strain illustrates some MIC variability within one dilution for all media, with the same modal MIC value across media. MIC read-out is clear, with no trailing or ambiguity.
(D) PA1562: This strain illustrates MIC variability within one dilution and the appearance of skipped wells. MIC read-out is clear, with no trailing or ambiguity. Skipped wells were discounted because inhibition of growth is reproduced in other lanes.
(E) PA1568: This strain illustrates MIC variability within one dilution and the appearance of skipped wells. MIC read-out is clear, with no trailing or ambiguity. Skipped wells were discounted because inhibition of growth is reproduced in other lanes.
(F) KP549: This strain illustrates MIC reproducibility across wells and days for each medium but shows different MIC values for different sources of broth. The skipped wells were discounted because inhibition of growth is reproduced in other lanes. This strain exhibits trailing in several media complicating MIC endpoint determinations.
(G) AB126: This strain illustrates trailing across all media and less reproducibility in MIC values between the plates. Despite applying the revised CLSI reading guidance, discrepant MIC endpoints are recorded.
(H) AB NCTC13301: This strain illustrates reproducible trailing across plates, days, and media. Application of the revised reading guidelines (MIC is recorded at the first well with a button size of ≤1 mm) results in different MIC values across media.
(I) AB148: This strain illustrates reproducible trailing and an unusual growth pattern (“donut” pattern). MIC is determined when growth reduction reaches ≥80%.

1-A

Strain: *Escherichia coli* EC461 [CTX-M-15, CMY-2] – historical BD-BBL MIC=1 µg/mL
Note: This strain illustrates MIC reproducibility across wells and days for each medium but shows different MIC values between sources of broth.
MIC read-out is clear, with no trailing or ambiguity.

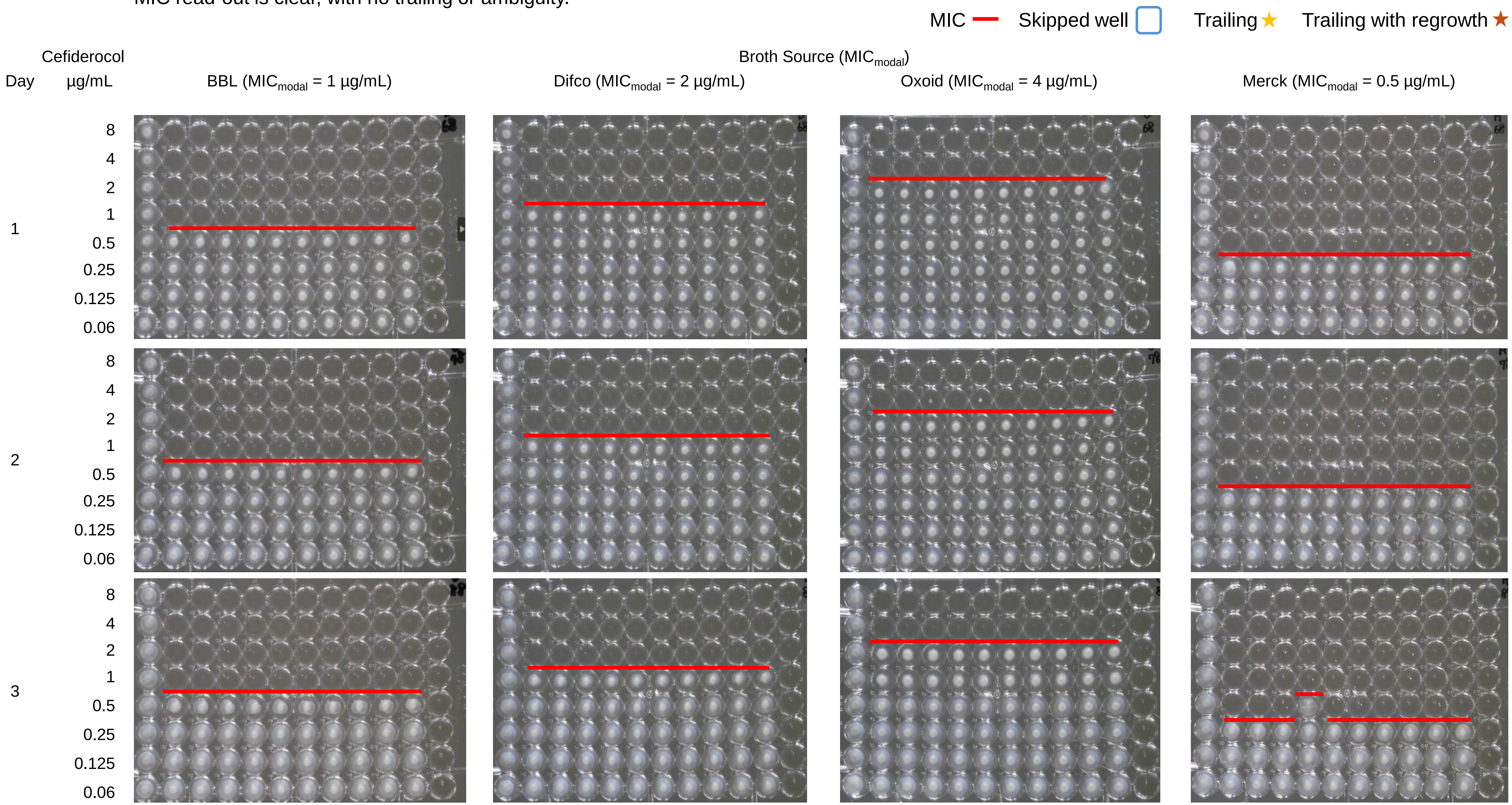

1-B

Strain: *Klebsiella pneumoniae* KP532 [information not available] – historical BD-BBL MIC=4 µg/mL

Note: This strain illustrates variability within one dilution for some media, but the same MIC across days for each medium. MIC values are different for different media. MIC read-out is clear, with no trailing or ambiguity.

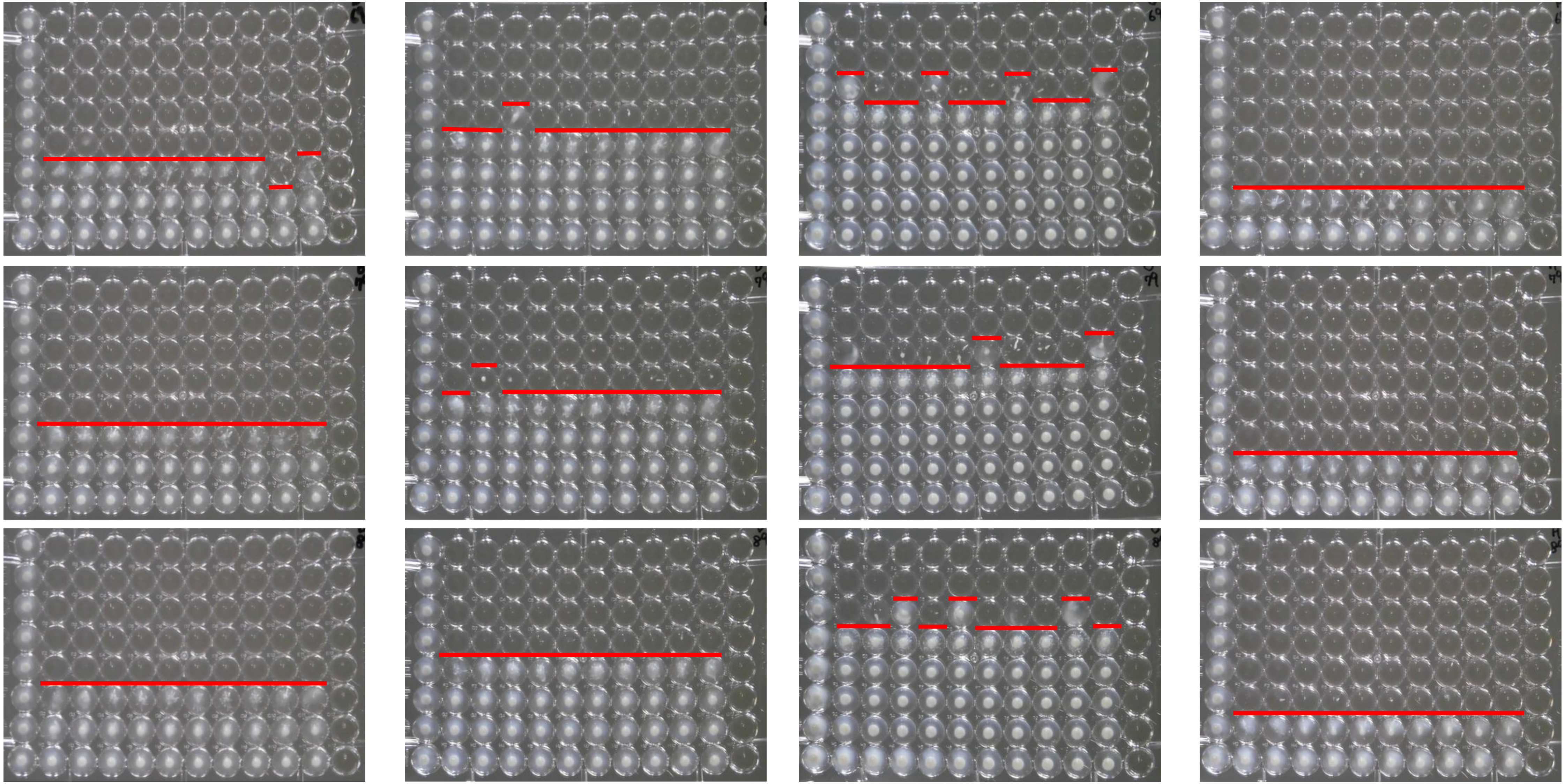

1-C

Strain: *Escherichia coli* EC466 [TEM-OSBL, CMY-2] – historical BD-BBL MIC=2 µg/mL
Note: This strain illustrates some MIC variability within one dilution for all media, with the same modal MIC across all media.
MIC read-out is clear, with no trailing or ambiguity.

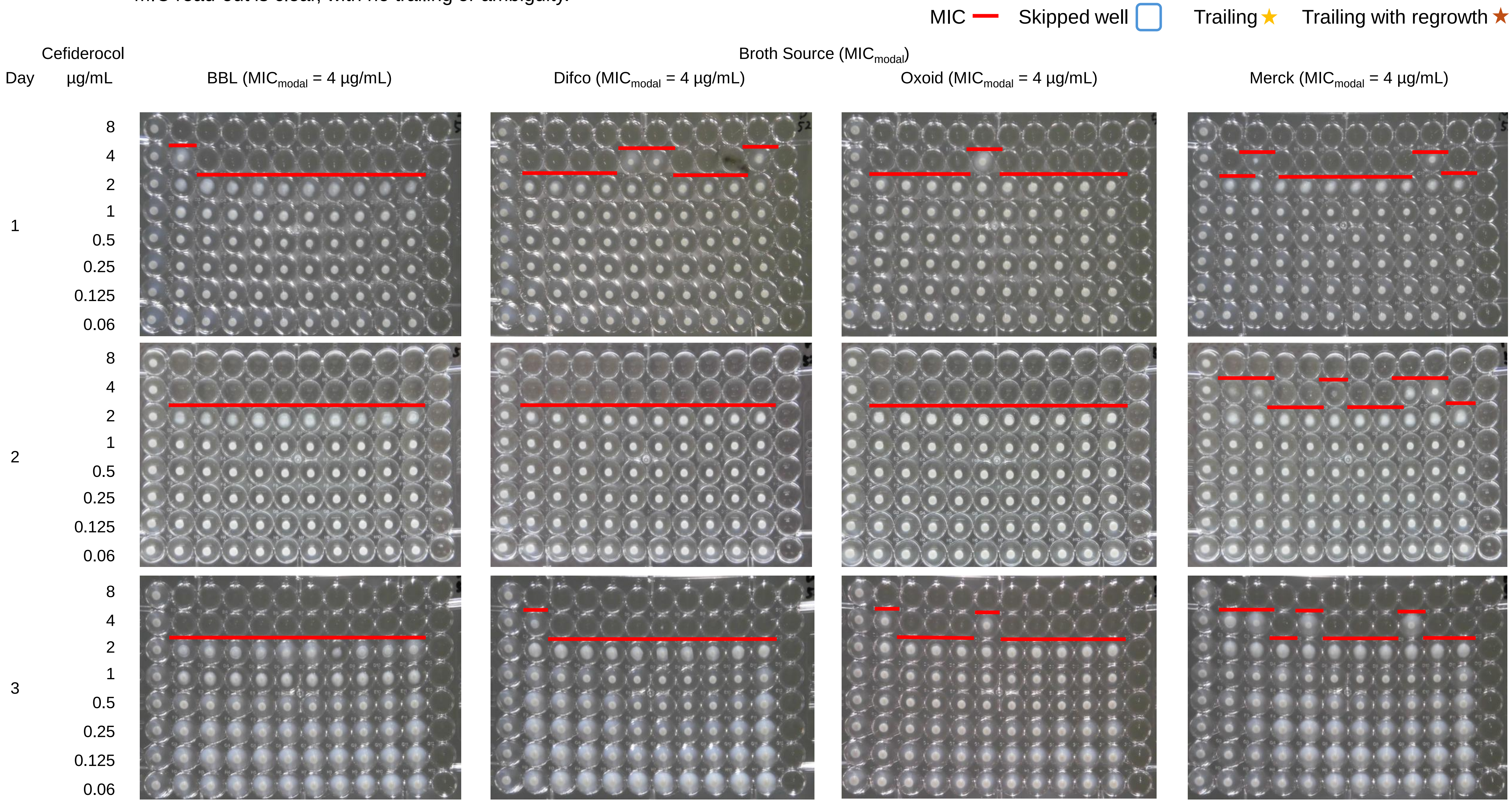

1-D

Strain: *Pseudomonas aeruginosa* PA1562 [PER-1] – historical BD-BBL MIC=1 µg/mL

Note: This strain illustrates MIC variability within one dilution and the appearance of skipped wells. MIC read-out is clear, with no trailing or ambiguity. Skipped wells were discounted here because inhibition of growth is reproduced in other lanes.

MIC — Skipped well ☐ Trailing ★ Trailing with regrowth ★

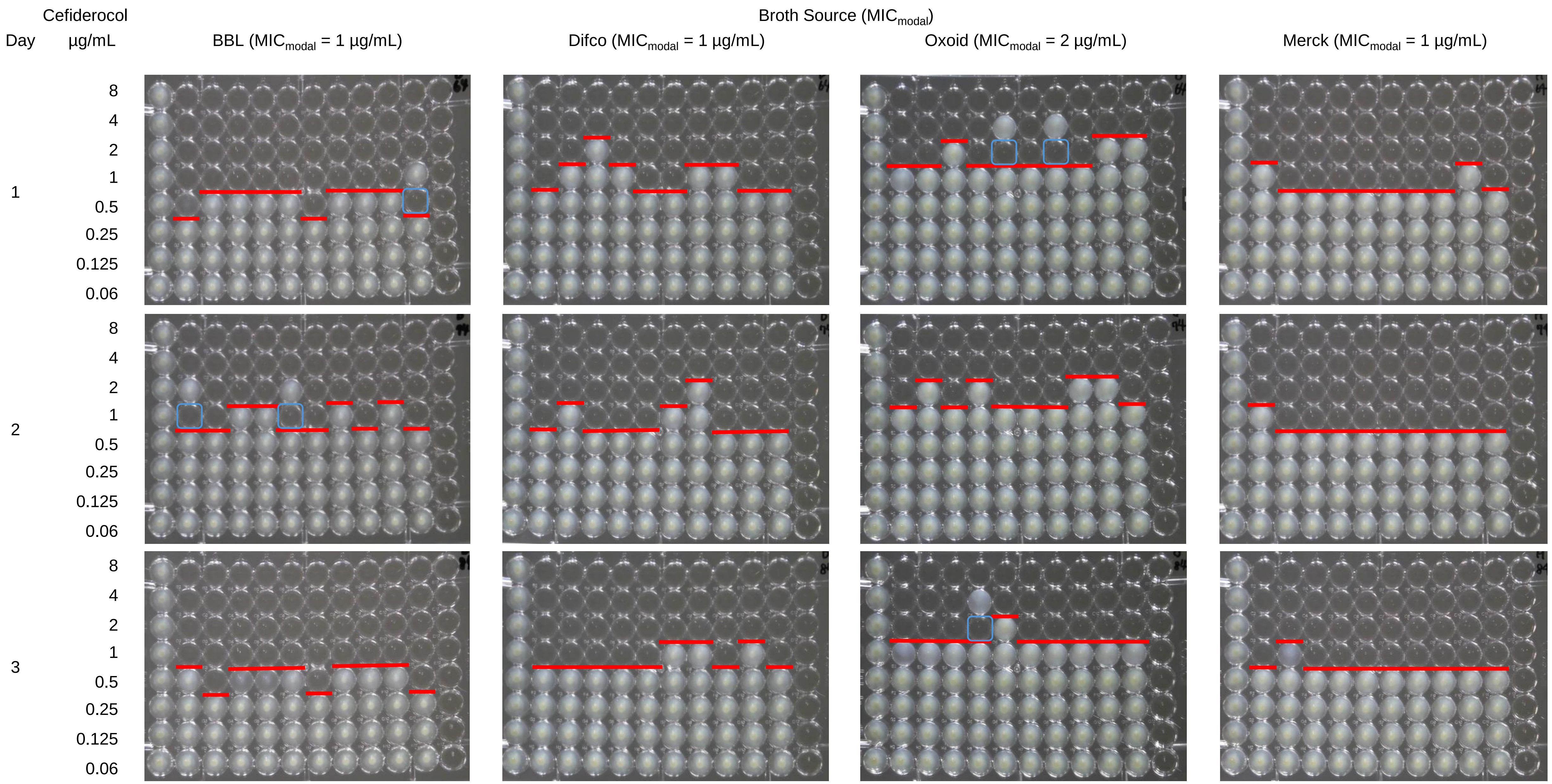

1-E

Strain: *Pseudomonas aeruginosa* PA1568 [VIM-2] – historical BD-BBL MIC=1 µg/mL
Note: This strain illustrates MIC variability within one dilution and the appearance of skipped wells. MIC read-out is clear, with no trailing or ambiguity. Skipped wells were discounted because inhibition of growth is reproduced in other lanes.

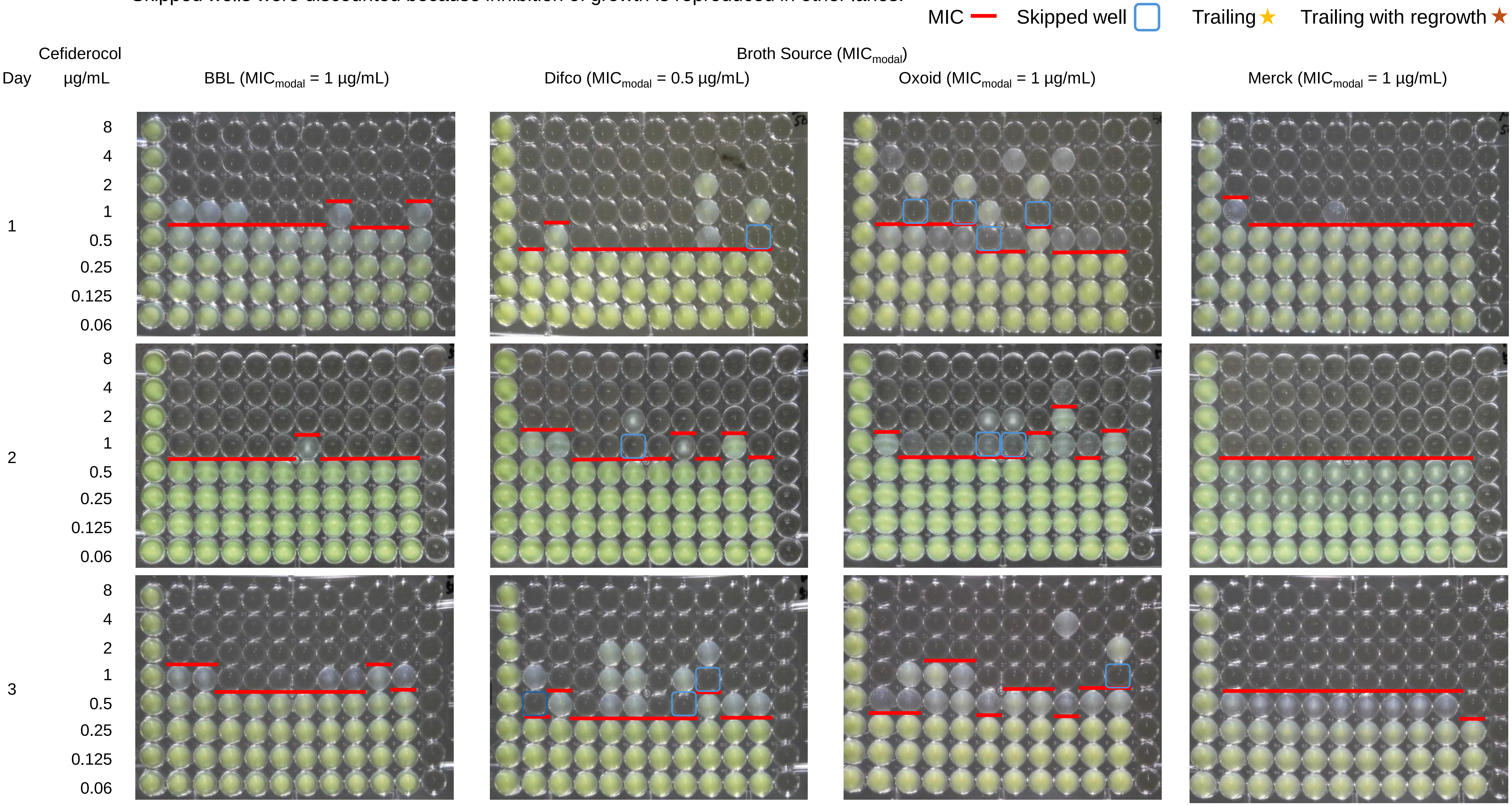

1-F

Strain: *Klebsiella pneumoniae* KP549 [SHV-12, TEM-OSBL] – historical BD-BBL MIC=16 µg/mL
Note: This strain illustrates MIC reproducibility across wells and days for each medium but shows different MIC values for different sources of broth.
The skipped wells were discounted because inhibition of growth is reproduced in other lanes.
This strain exhibits trailing in several media complicating MIC endpoint determinations.

MIC — Skipped well □ Trailing ★ Trailing with regrowth ★

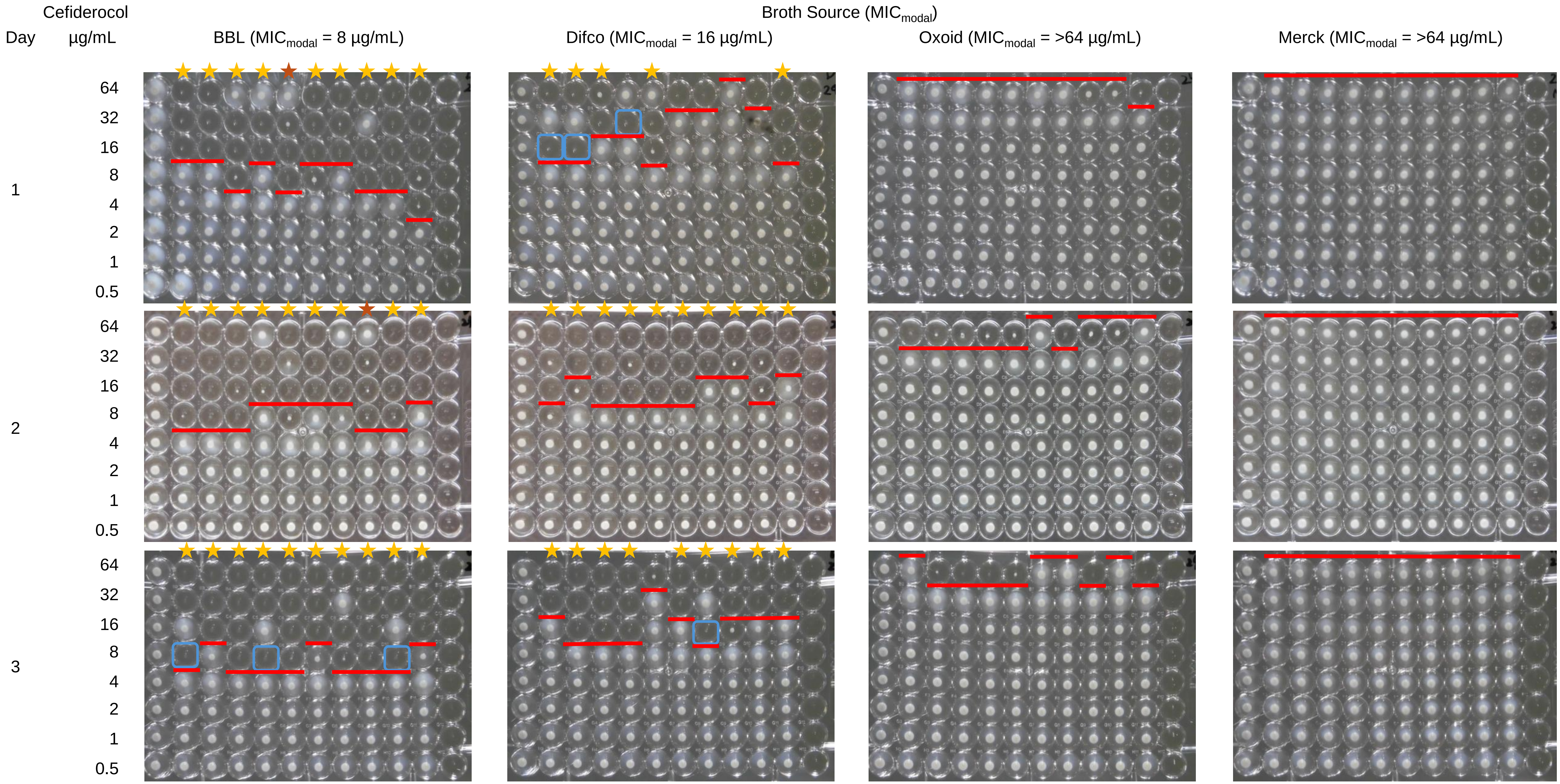

1-G

Strain: *Acinetobacter baumannii* AB126 [ADC-33, OXA-82] – historical BD-BBL MIC=8 µg/mL

Note: This strain illustrates trailing across all media and less reproducibility in MIC values between the plates. Despite applying the revised CLSI reading guidance, discrepant MIC endpoints are recorded.

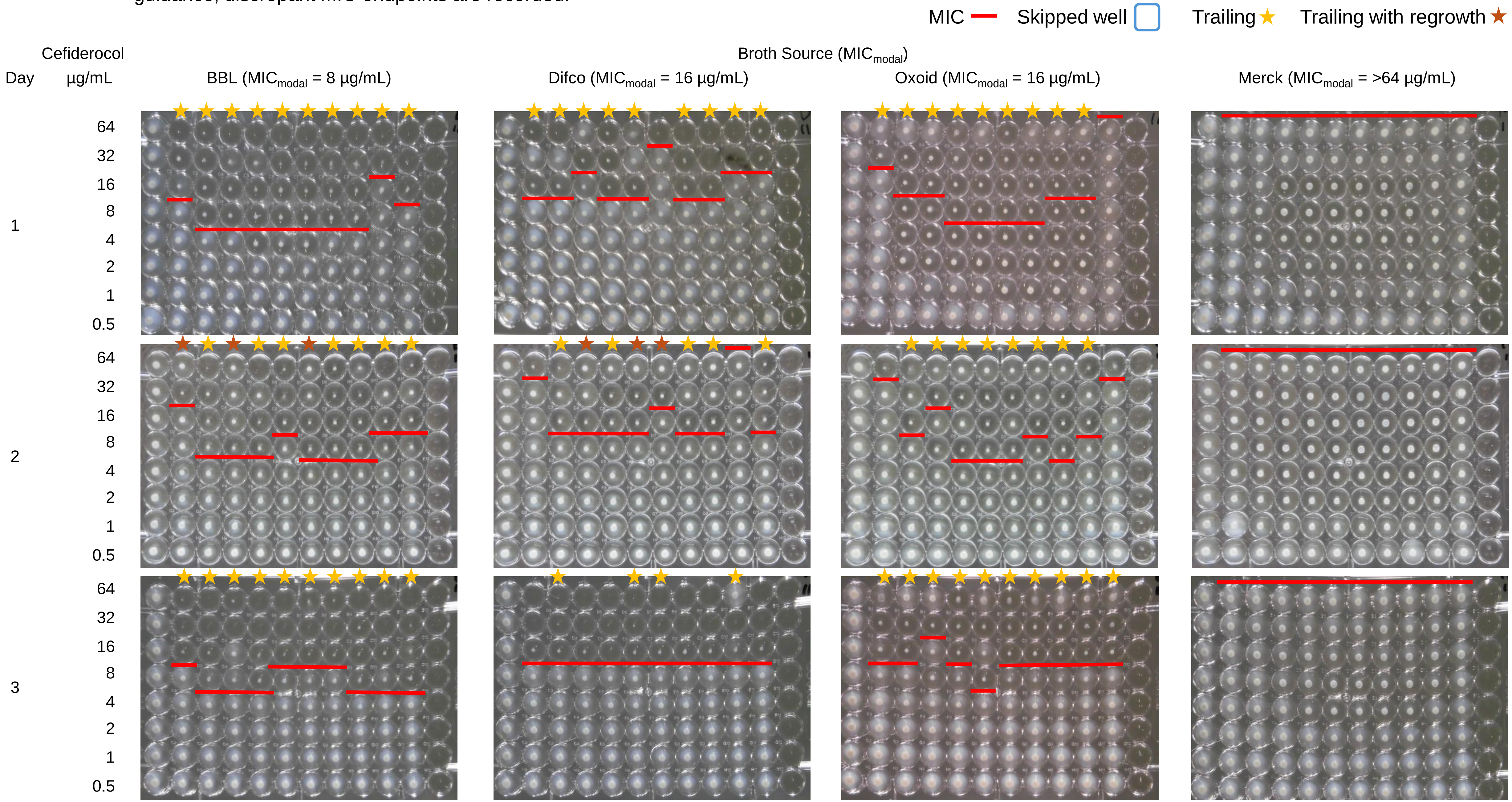

1-H

Strain: *Acinetobacter baumannii* NCTC13301 [OXA-23] – historical BD-BBL MIC= not available

Note: This strain illustrates reproducible trailing across plates, days and media. Application of the revised reading guidelines (MIC is recorded at the first well with a button size of ≤1 mm) results in different MIC values across media.

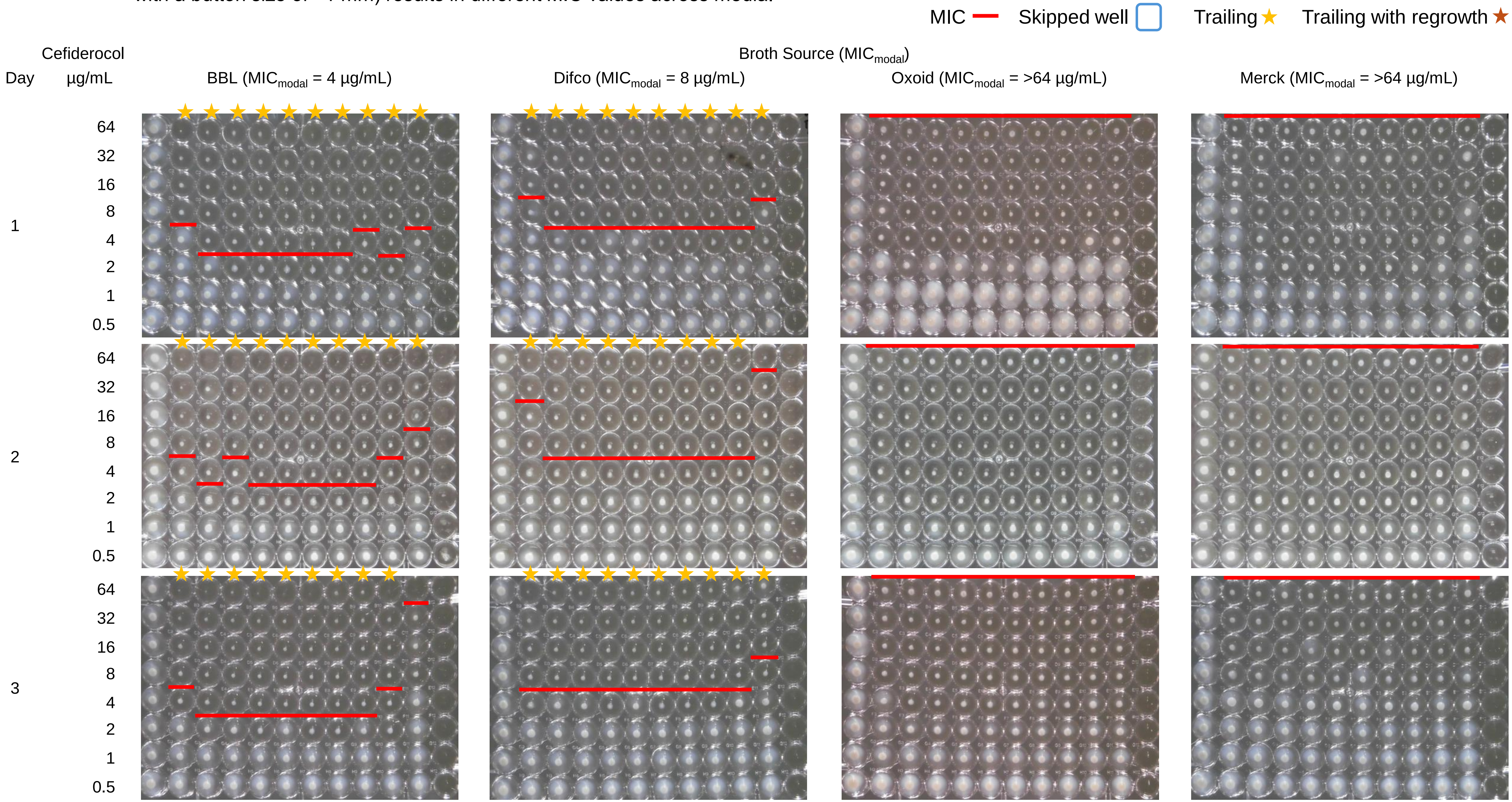

1-I

Strain: *Acinetobacter baumannii* AB148 [TEM-OSBL] – historical BD-BBL MIC=0.5 µg/mL

Note: This strain illustrates a reproducible trailing and an unusual growth pattern ("donut" pattern). MIC is determined when growth reduction reaches ≥80%.

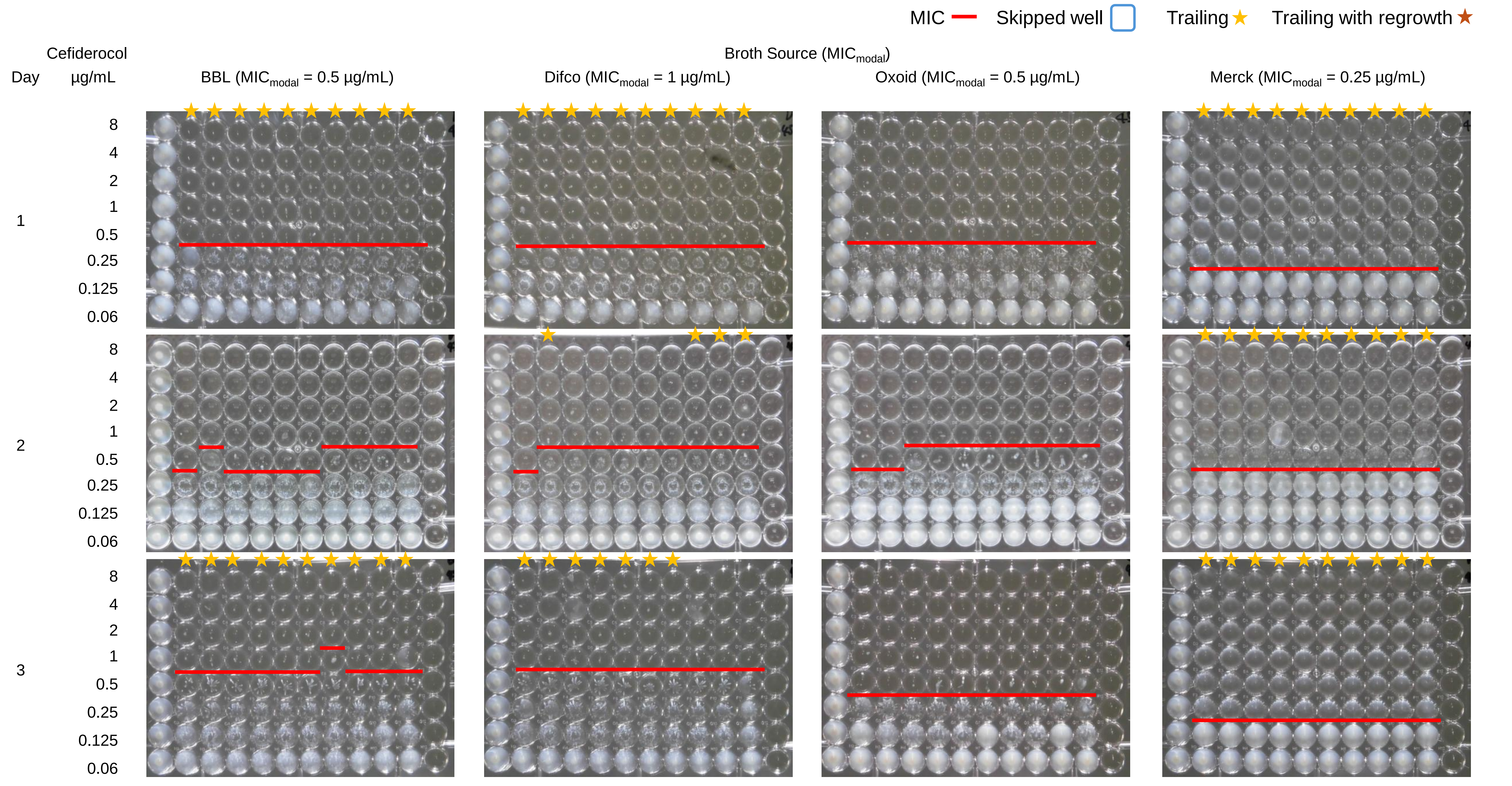

Supplement: Fig. S1 — MIC determinations for E. coli, K. pneumoniae, P. aeruginosa, and A. baumannii strains using ID-CAMHB sourced from BD-BBL, BD-Difco, Oxoid, and Merck. [file jcm.00471-25-s0001.pdf]
