# Supplementary material for: The impact of commercially available media on cefiderocol susceptibility testing by broth microdilution method
Source: J Clin Microbiol. 2025 Aug 20;63(9):e00471-25. doi: 10.1128/jcm.00471-25 (PMC12421808; doi:10.1128/jcm.00471-25)
Supplement: Fig. S2 — Summary of the preparation of the iron-depleted cation-adjusted Mueller–Hinton broth. [file jcm.00471-25-s0002.pdf]

Supplemental FIG S2 Summary of the preparation of the iron-depleted cation-adjusted Mueller-Hinton broth

PREPARATION OF

Iron-Depleted Cation-Adjusted Mueller–Hinton Broth

1
Adding Chelex® 100

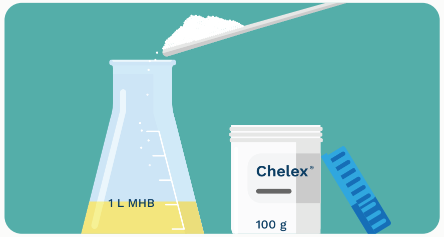

Take 1 L autoclaved MHB cooled down to room temperature.

Add 100 g Chelex® 100 chelating resin, analytical grade [100–200 mesh, Na^{+} form].

2
6-h stir + filtering

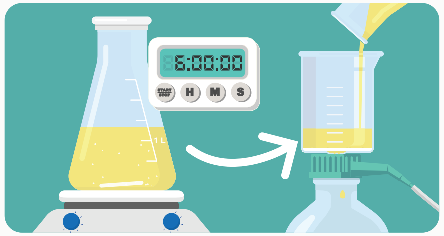

Stir at room temperature for at least 6 hours.

Filter using 0.2 μm pore-size filter to remove resin.

3
pH adjustment

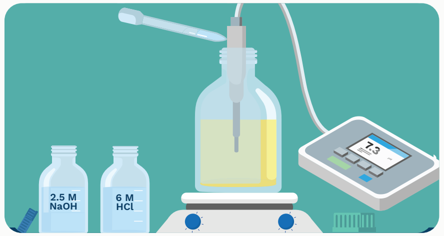

Adjust pH to 7.2–7.4 by using 6 M HCl or 2.5 M NaOH.

4
Cation adjustment

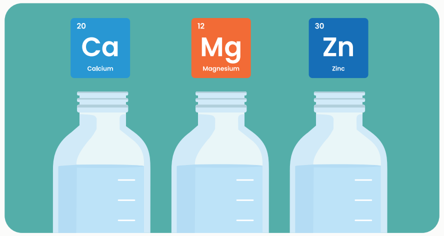

Add Ca^{2+} (final 20–25 μg/mL).

Add Mg^{2+} (final 10–12.5 μg/mL).

Add Zn^{2+} (final 0.5–1 μg/mL).

5
pH adjustment + filtering

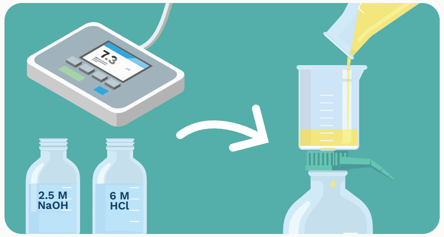

Adjust pH to 7.2–7.4 by using 6 M HCl or 2.5 M NaOH.

Filter using 0.2 μm pore-size filter.

6
Iron concentration measurement

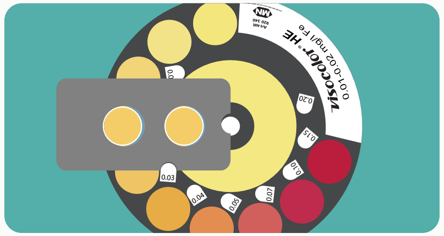

Measure iron concentration (final ≤0.03 μg/mL).

7
Storage at 4 °C

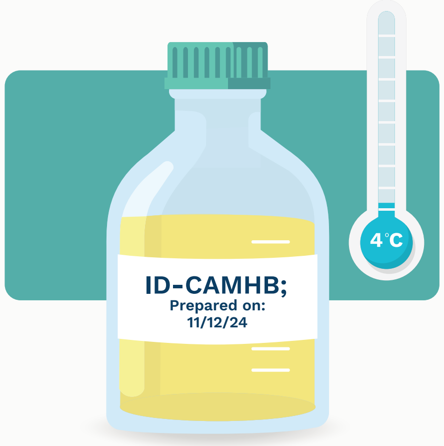

Avoid iron contamination

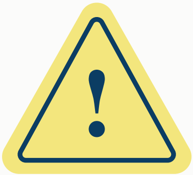

To avoid contamination with iron during the preparation of the iron-depleted cation-adjusted Mueller-Hinton broth, use distilled or deionized water, plastic instead of metal spatulas, Teflon™-coated magnetic bars, and high-purity reagents.
