# Supplementary material for: The impact of commercially available media on cefiderocol susceptibility testing by broth microdilution method
Source: J Clin Microbiol. 2025 Aug 20;63(9):e00471-25. doi: 10.1128/jcm.00471-25 (PMC12421808; doi:10.1128/jcm.00471-25)
Supplement: Supplemental Methods — Protocol of inductively coupled plasma atomic emission spectrometry (ICP-AES) and inductively coupled plasma mass spectrometry (ICP-MS). [file jcm.00471-25-s0003.pdf]

Supplemental METHODS Protocol of inductively coupled plasma atomic emission spectrometry (ICP-AES) and inductively coupled plasma mass spectrometry (ICP-MS).

1. ICP-AES (INDUCTIVELY COUPLED PLASMA ATOMIC EMISSION SPECTROMETRY)

1.1 Testing facility

Shionogi & Co., Ltd, Osaka, Japan

1.2 Preparation of standard solution

1) Put about 15 mL of 0.5% nitric acid in a DigiTube (AnalytiChem Canada, Inc.) in advance.

2) Add exactly 500 µL of multi-element mixed standard solution (1000 mg/L) to the DigiTube, and then add more 0.5% nitric acid to make a total of 20 mL; use this solution as the standard stock solution.

3) Accurately measure out 0, 10, 20, 40, and 100 µL, respectively, of the standard stock solution, add 250 µL of the internal standard stock solution to each, and then add 0.5% nitric acid to make a total of 25 mL for each, and use these solutions as the standard solutions of 0, 0.010, 0.020, 0.040, and 0.100 µg/mL.

1.3 Preparation of sample solution

I) Add 1000 mg of sample to a DigiTube.

2) Add approximately 25 mL of 0.5% nitric acid to dissolve, then add exactly 500 µL of the internal standard stock solution.

3) Add 0.5% nitric acid to make 50 mL, and use this as the sample solution.

1.4 Test conditions and settings

Apparatus: Shimadzu ICPE-9000 Multitype Spectrometer

Metals and wavelengths: Al 167.081 nm, Ca 183.801 nm, Co 228.616 nm,
Cr 205.552 nm, Cu 224.700 nm, Fe 238.204 nm, K 766.490 nm, Mg 285.213 nm,
Mn 260.569 nm, Ni 231.604 nm, Y 3 71.030 nm, Zn 206.200 nm

Sample introduction system
- Nebulizer: coaxial nebulizer (uptake 1 mL/min)
- Spray chamber: cyclone type
- Sample introduction amount: 1.0 mL/min

Plasma conditions
- Frequency: 27.12 MHz
- High frequency power: 1.2 kW
- Plasma gas: 14 L/min
- Auxiliary gas: 1.2 L/min
- Carrier gas: 0.7 L/min

Spectrometer conditions
- Observation direction: axial

- Measurement sensitivity: high
- Illumination time: 20 s
- Number of integrations: 3
- Number of pixels: 3
- Background correction: 2 points

1.5 System suitability

1.5.1 System performance

1) For the standard solution, determine the emission intensity ratio (E/Y) of each element (E) to yttrium (Y).

2) Create a calibration curve at five levels with the concentration of the standard solution on the horizontal axis and the emission intensity ratio (E/Y) on the vertical axis.

3) The correlation coefficient of the calibration curve for each element is 0.99 or more.

1.6 Calculation formula

The concentration of each metal (ppm) = $\frac{(Rt-b)}{a} \times \frac{1}{W} \times c$

- *a*: slope of calibration curve
- *b*: y-intercept of calibration curve
- *c*: dilution ratio 50×
- *Rt*: intensity ratio of each metal to yttrium in the sample solution
- W: sample weight (g)

1.7 Reagents and test solutions

Other than those specified separately, use the reagents, test solutions, and standards of the Japanese Pharmacopoeia. Note that the water used for sample preparation should have an electrical conductivity of 18 MΩ·cm or more.

- Multielement mixed standard solution (1000 mg/L): ICP-multielement standard solution IV (Merck, USA)
- Internal standard stock solution: accurately extract 500 μL of yttrium standard solution (1000 mg/L) and add 0.5% nitric acid to make 20 mL
- Yttrium (Y) standard solution (1000 mg/L): 1000 mg/L solution for atomic absorption spectrometry or equivalent
- 0.5% nitric acid: add 5 mL of nitric acid to 1000 mL of water
- Nitric acid: ultramicroanalysis grade or equivalent
- Sulfuric acid: atomic absorption spectrometry grade or equivalent

2. ICP-MS (INDUCTIVELY COUPLED PLASMA MASS SPECTROMETRY)

2.1 Testing facility

Shionogi Pharma Co., Ltd, Osaka, Japan

2.2 Reagents and test solutions

- Water: electrical conductivity of 18 MΩcm or more
- Nitric acid: for measuring trace metals (Kanto Chemical, Tokyo, Japan)
- Magnesium standard solution (1000 mg/L): Wako Pure Chemical (Osaka, Japan)
- Calcium standard solution (1000 mg/L): Wako Pure Chemical
- Iron standard solution (1000 mg/L): Wako Pure Chemical
- Zinc standard solution (1000 mg/L): Wako Pure Chemical
- Yttrium standard solution (1000 mg/L): for chemical analysis (Kanto Chemical)
- Internal standard solution: accurately measure 10 µL of yttrium standard solution (1000 mg/L), add 1 mL of nitric acid and water to make 100 mL

2.3 Preparation of standard solutions

1) Add exactly 250 µL of each standard solution (1000 mg/L) to a 50 mL measuring flask, then add 500 µL of nitric acid and water to make 50 mL (standard stock solution: 5 mg/L).

2) Add 0, 50, 100, 500, 1000, 5000 µL of the standard stock solution accurately to each 50 mL measuring flask, then add 500 µL of nitric acid and water to make 50 mL (standard solutions 0, 5, 10, 50, 100, 500 ng/mL).

3) The calibration curve range is as follows, and the calibration curve should be selected according to the concentration of the sample solution.

- Calibration curve 1: 0, 5, 10, 50 ng/mL
- Calibration curve 2: 0, 5, 10, 50, 100, 500 ng/mL

2.4 Preparation of sample solutions

2.5× diluted solution

- Put 8000 µL of the shaken sample in a sterile tube, add 200 µL of nitric acid and water to make 20 mL. Record the weight of the sample.

10× diluted solution

- Put 2000 µL of the shaken sample in a sterile tube, add 200 µL of nitric acid and water to make 20 mL. Record the weight of the sample.

100× diluted solution

- Put 2000 µL of the 10× diluted solution, add 200 µL of nitric acid and water to make 20 mL. Record the weight of the sample.

2.5 Apparatus and measured metals

Apparatus: Agilent Technologies 7700× ICP-MS

Measured metals and mass: Mg 24 m/z, Ca 44 m/z, Fe 56 m/z, Zn 66 m/z, Y (internal standard substance) 89 m/z

The internal standard solution is not added directly to the standard solution or sample solution, but is added automatically online.
