# Supplementary material for: The impact of commercially available media on cefiderocol susceptibility testing by broth microdilution method
Source: J Clin Microbiol. 2025 Aug 20;63(9):e00471-25. doi: 10.1128/jcm.00471-25 (PMC12421808; doi:10.1128/jcm.00471-25)
Supplement: Table S2 — Unadjusted cation concentrations in different brands of cation-adjusted Mueller–Hinton broth measured using ICP-AES after 0 and 24 h of chelation. [file jcm.00471-25-s0005.pdf]

Supplemental TABLE S2 Unadjusted cation concentrations in different brands of cation-adjusted Mueller–Hinton broth measured using inductively coupled plasma atomic emission spectrometry (ICP-AES) after 0 and 24 h of chelation

	**Cation concentration (μg/mL) measured by ICP-AES at the given chelation time points[a]**																					
	**Mg^{2+}**		**Al^{3+}**		**K^{+}**		**Ca^{2+}**		**Cr^{2+}**		**Mn^{2+}**		**$Fe^{2+/3+}$**		**Co^{2+}**		**Ni^{2+}**		**Cu^{2+}**		**Zn^{2+}**	
Broth manufacturer/ details	**0 h**	**24 h**	**0 h**	**24 h**	**0 h**	**24 h**	**0 h**	**24 h**	**0 h**	**24 h**	**0 h**	**24 h**	**0 h**	**24 h**	**0 h**	**24 h**	**0 h**	**24 h**	**0 h**	**24 h**	**0 h**	**24 h**
BD-BBL MHIIB, Lot no. 2089488	13.007	0.050	0.0100	ND	97.5	90.2	21.429	0.180	0.020	0.040	0.020	0.010	0.540	0.030	0.010	0.010	0.060	0.010	0.030	ND	1.000	ND
BD-BBL MHIIB, Lot no. 1151190	12.147	0.020	0.260	ND	92.7	79.6	24.109	ND	0.020	0.030	0.020	0.010	0.310	0.020	0.010	0.010	0.040	0.020	0.010	0.040	0.980	ND
BD-BBL MHB, Lot no. 2030884	2.490	0.040	0.090	ND	96.3	84.1	19.529	0.100	0.020	0.020	0.020	0.010	0.240	0.020	0.010	ND	0.030	0.030	0.030	0.010	1.020	ND
Difco MHB, Lot no. 1089531	3.559	0.030	0.140	ND	149.3	125.5	3.320	0.180	0.020	0.040	0.020	0.010	0.340	0.030	0.020	0.010	0.040	0.020	0.050	ND	0.360	ND
Merck MHB, Lot no. VM235893	4.269	0.030	0.250	ND	119.4	85.5	11.569	0.040	0.020	ND	0.030	0.010	0.310	0.020	0.020	ND	0.010	ND	0.010	0.010	0.770	ND
Oxoid MHB, Lot no. 1162586	6.409	0.020	0.070	0.100	212.7	172.3	4.900	ND	0.040	0.070	0.030	0.010	0.590	0.060	0.010	ND	0.080	0.010	0.040	0.010	0.280	ND

MHB, Mueller–Hinton broth; MHIIB, Mueller–Hinton II broth; ND, not detectable.

[a]0 h and 24 h are the chelation durations. “0 h” corresponds to cation-adjusted MHB (MHIIB) or MHB. “24 h” corresponds to iron-depleted MHB (other cations were unadjusted).
